# Nucleosomes around a mismatched base pair are excluded via an Msh2-dependent reaction with the aid of SNF2 family ATPase Smarcad1

**DOI:** 10.1101/gad.310995.117

**Published:** 2018-06-01

**Authors:** Riki Terui, Koji Nagao, Yoshitaka Kawasoe, Kanae Taki, Torahiko L. Higashi, Seiji Tanaka, Takuro Nakagawa, Chikashi Obuse, Hisao Masukata, Tatsuro S. Takahashi

**Affiliations:** 1 Graduate School of Science, Osaka University, Toyonaka, Osaka 560-0043, Japan;; 2 Faculty of Science, Kyushu University, Nishi-ku, Fukuoka 819-0395, Japan;; 3 Graduate School of Life Science, Hokkaido University, Sapporo, Hokkaido 060-0810, Japan;; 4 Division of Microbial Genetics, National Institute of Genetics, Mishima, Shizuoka 411-8540, Japan;; 5 School of Environmental Science and Engineering, Kochi University of Technology, Kami-city, Kochi 782-8502, Japan

**Keywords:** mismatch repair, chromatin, nucleosome, *Xenopus* egg extract, yeast

## Abstract

Post-replicative correction of replication errors by the mismatch repair (MMR) system is critical for suppression of mutations. Although the MMR system may need to handle nucleosomes at the site of chromatin replication, how MMR occurs in the chromatin environment remains unclear. Here, we show that nucleosomes are excluded from a >1-kb region surrounding a mismatched base pair in *Xenopus* egg extracts. The exclusion was dependent on the Msh2–Msh6 mismatch recognition complex but not the Mlh1-containing MutL homologs and counteracts both the HIRA- and CAF-1 (chromatin assembly factor 1)-mediated chromatin assembly pathways. We further found that the Smarcad1 chromatin remodeling ATPase is recruited to mismatch-carrying DNA in an Msh2-dependent but Mlh1-independent manner to assist nucleosome exclusion and that Smarcad1 facilitates the repair of mismatches when nucleosomes are preassembled on DNA. In budding yeast, deletion of *FUN30*, the homolog of Smarcad1, showed a synergistic increase of spontaneous mutations in combination with *MSH6* or *MSH3* deletion but no significant increase with *MSH2* deletion. Genetic analyses also suggested that the function of Fun30 in MMR is to counteract CAF-1. Our study uncovers that the eukaryotic MMR system has an ability to exclude local nucleosomes and identifies Smarcad1/Fun30 as an accessory factor for the MMR reaction.

## Abstract (precis)

Here, Terui et al. studied the mechanisms underlying chromatin remodeling that occurs during MMR. They show that the eukaryotic MMR system has an ability to exclude local nucleosomes and identify Smarcad1/Fun30 as an accessory factor for the MMR reaction.

Precise DNA replication relies on both the fidelity of DNA synthesis by DNA polymerases and post-replicative correction of replication errors by the mismatch repair (MMR) system. MMR is critical for the suppression of tumorigenesis, as inactivation of MMR genes gives rise to a high risk of hereditary and sporadic cancers (Lynch et al. 2015). Post-replicative MMR occurs through the following steps: mismatch recognition, searching for strand discrimination signals, nicking on the newly synthesized DNA strand, degradation of the strand, and resynthesis of the removed segment (for reviews, see Iyer et al. 2006; Jiricny 2013; Kunkel and Erie 2015). Replication errors are recognized by two Msh2-containing complexes: MutSα (Msh2–Msh6) and MutSβ (Msh2–Msh3). The substrate specificities of the two complexes are partially overlapped, especially on small insertion/deletion loops (IDLs), although MutSα exhibits a preference for base–base mismatches and small IDLs, and MutSβ exhibits a preference for large IDLs (Marsischky et al. 1996; Genschel et al. 1998). In mammalian cells, MutSα is much more abundant than MutSβ and is primarily responsible for repair of base–base mismatches and small IDLs (Drummond et al. 1997; Genschel et al. 1998; Marra et al. 1998). Mismatch-bound MutSα and MutSβ change their conformation to sliding clamps, recruit the MutLα endonuclease (Mlh1–Pms2 in vertebrates), and translocate along DNA (for reviews, see Iyer et al. 2006; Jiricny 2013; Lee et al. 2014; Kunkel and Erie 2015). This reaction is likely required for the search for strand discrimination signals such as ssDNA breaks, repair intermediates of ribonucleotides embedded in the leading strand, and DNA-bound proliferating cell nuclear antigen (PCNA) (Holmes et al. 1990; Thomas et al. 1991; Pluciennik et al. 2010; Ghodgaonkar et al. 2013; Lujan et al. 2013; Kawasoe et al. 2016). Communication between MutLα, MutSα/MutSβ, and DNA-bound PCNA induces strand-specific nicking by the MutLα endonuclease (Kadyrov et al. 2006; Pluciennik et al. 2010, 2013). A mismatch-containing segment is then degraded mainly by Exonuclease 1 (Exo1) (Tishkoff et al. 1997; Amin et al. 2001). Finally, DNA polymerases resynthesize the degraded segment to complete the repair reaction.

Most DNA transactions occurring on chromatin require the movement, exchange, or displacement of nucleosomes (for reviews, see Ransom et al. 2010; Narlikar et al. 2013; Polo and Almouzni 2015). Histone chaperone CAF-1 (chromatin assembly factor 1) and HIRA support DNA synthesis-coupled and synthesis-independent chromatin assembly, respectively (Smith and Stillman 1989; Gaillard et al. 1996; Ray-Gallet et al. 2002). FACT (facilitates chromatin transcription) promotes the exchange of histones, particularly H2A–H2B dimers, at the site of transcription, replication, and repair (for review, see Formosa 2012). Some DNA repair reactions are assisted by a specific class of adenosine triphosphate (ATP)-dependent chromatin remodeling enzyme (chromatin remodeler) (for review, see Narlikar et al. 2013). An example is SNF2-type chromatin remodeling enzyme Smarcad1, which facilitates long-range resection of double-strand break ends in the context of chromatin in both humans and yeast (Chen et al. 2012; Costelloe et al. 2012; Eapen et al. 2012; Densham et al. 2016). In addition to double-strand break end processing, Smarcad1 is involved in heterochromatin silencing (Neves-Costa et al. 2009; Rowbotham et al. 2011; Stralfors et al. 2011; Taneja et al. 2017). This factor is also enriched on the nascent DNA at the replication fork (Rowbotham et al. 2011; Sirbu et al. 2013) and is physically associated with Msh2-containing complexes (Okazaki et al. 2008; Rowbotham et al. 2011; Chen et al. 2016), yet the significance of these observations remains to be elucidated.

Accumulating evidence suggests that nucleosomes are assembled immediately behind the replication fork (McKnight and Miller 1977; Sogo et al. 1986; Lucchini and Sogo 1995; Shibahara and Stillman 1999; Smith and Whitehouse 2012), and thus post-replicative MMR may need to contend with nucleosomes to carry out its function in cells. On the one hand, both eukaryotic MutLα and bacterial MutL form a large proteinaceous ring that can rapidly diffuse along DNA, and eukaryotic MutLα can hop over nucleosomes (Gorman et al. 2010; Liu et al. 2016). Therefore, chromatin structure may not prevent the communication between MutLα and PCNA. MutSβ can also jump over nucleosomes (Brown et al. 2016), and the MutSβ-dependent step in MMR could also function on chromatin. On the other hand, both single-molecule and biochemical studies demonstrated that nucleosomes are inhibitory for diffusion of MutSα along DNA (Gorman et al. 2010; Brown et al. 2016) and the MutSα-dependent MMR reaction (Li et al. 2009, 2013; Schopf et al. 2012). A possible means to assist a MutSα-dependent reaction on chromatin may simply be to localize it close to the replication fork, where nucleosomes must be transiently disassembled. MutSα is tethered to the replication machinery through its conserved PCNA-interacting motif (Kleczkowska et al. 2001; Hombauer et al. 2011; Haye and Gammie 2015) and to nucleosomes containing K36-methylated histone H3 through its PWWP motif, found in vertebrates (Li et al. 2013). Another possible means is to exclude or disassemble nucleosomes near replication errors. In vitro reconstitution studies have shown that human MutSα exhibits chromatin remodeling activity (Javaid et al. 2009) and that it competes with CAF-1-dependent chromatin assembly (Kadyrova et al. 2011; Schopf et al. 2012; Rodriges Blanko et al. 2016). Two chromatin-related factors, HMGB1 and regulatory factor X, are also reported to stimulate MMR in vitro (Yuan et al. 2004; Zhang et al. 2008), although their involvement in MMR in vivo has not been established. Despite the progress, however, how nucleosomes are handled by the MMR machinery during the repair reaction remains largely elusive. Furthermore, while many DNA repair reactions are assisted by chromatin remodelers and histone chaperones (Ransom et al. 2010; Narlikar et al. 2013; Polo and Almouzni 2015), it remains unclear whether the MMR system receives assistance from such factors in vivo.

Using *Xenopus* egg extracts as a model system, we studied how the MMR system handles nucleosomes after the recognition of a mismatch. We show here that nucleosomes around a mispaired base are efficiently excluded via an Msh2-dependent reaction. We further show that Smarcad1 is recruited to mismatch-carrying DNA depending on Msh2, assists nucleosome exclusion, and facilitates the repair of mismatches when nucleosomes are preassembled on DNA. Genetic experiments in yeast provide evidence that the homolog of Smarcad1 has a mutation suppressor function that is epistatic to Msh2 and antagonizes CAF-1. Our results reveal a dynamic interplay between MMR and chromatin and identify Smarcad1/Fun30 as a factor that assists the MMR reaction.

## Results

### Nucleosomes are excluded from a >1-kb region surrounding a mismatch in *Xenopus* egg extracts

**Figure 1.**
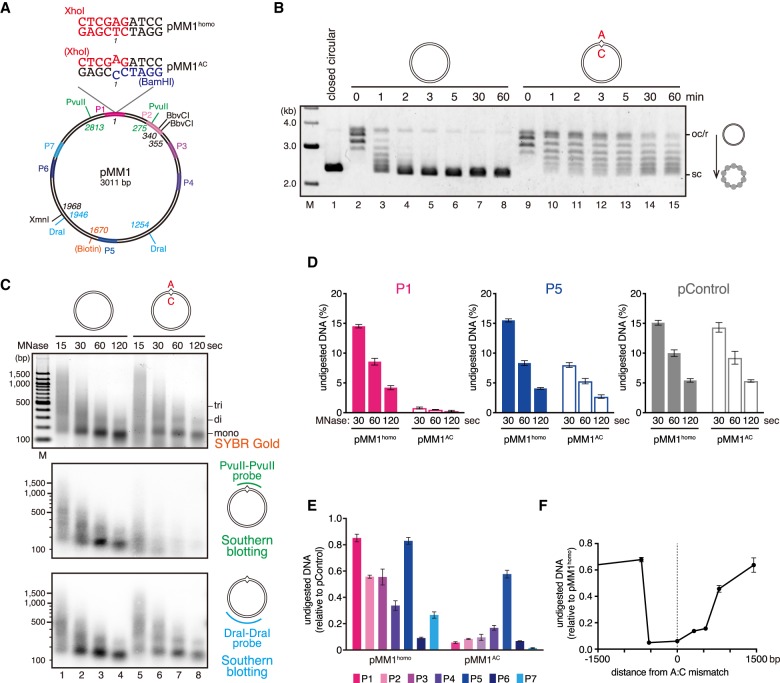
Nucleosomes are excluded from a >1-kb region surrounding a mismatch. (*A*) The DNA substrate used in this study. The 3011-base-pair (bp) DNA carries an A:T base pair (pMM1^homo^) or an A:C mispair (pMM1^AC^) at position 1. Positions of restriction enzyme sites used in this study, the site of biotin modification, and amplicons for quantitative PCR (qPCR) (P1: 2950–61, P2: 253–383, P3: 476–602, P4: 728–860, P5: 1498–1628, P6: 2266–2397, and P7: 2413–2537) are indicated. (*B*) Supercoiling assay in NPE. Covalently closed pMM1^homo^ (lanes *2*–*8*) or pMM1^AC^ (lanes *9*–*15*) was incubated in NPE and sampled at the indicated times. (Lane *1*) Supercoiled pMM1^homo^ purified from *Escherichia coli* was used as a size standard. (oc/r) Open circular or relaxed DNA; (sc) supercoiled DNA. (*C*) pMM1^homo^ (lanes *1*–*4*) or pMM1^AC^ (lanes *5*–*8*) was incubated in NPE for 60 min and digested by micrococcal nuclease (MNase). DNA samples stained with SYBR Gold (*top*) and Southern blotting with the PvuII–PvuII probe (*middle*) and the DraI–DraI probe (*bottom*) are shown. (*D*–*F*) The MNase assay described in *C* was repeated in the presence of a control plasmid (pControl), and undigested DNA was quantified by qPCR. The amount of DNA relative to the input (*D*) and normalized to pControl (*E*) and pMM1^homo^ (*F*) is presented. Mean ± one standard deviation (SD) is shown. *n* = 3.

To study MMR in the context of chromatin, we used extracts of *Xenopus* eggs, which efficiently recapitulate DNA synthesis, MMR (Olivera Harris et al. 2015; Kawasoe et al. 2016), and both DNA synthesis-independent (HIRA-mediated) (Ray-Gallet et al. 2002) and synthesis-coupled (CAF-1-mediated) chromatin assembly (Gaillard et al. 1996) in vitro. Deposition of a nucleosome induces approximately one compensatory positive supercoil in closed circular duplexes, and by relaxing this torsional strain, topoisomerase I reduces the linking number of a plasmid by one for each nucleosome assembled. Upon incubation in the nucleoplasmic extract (NPE) of *Xenopus* eggs (Walter et al. 1998), a 3.0-kb closed circular plasmid (pMM1^homo^) (Fig. 1A) became supercoiled within 2–3 min (Fig. 1B, lanes 2–6). Since NPE recapitulates the S-phase nuclear environment that does not allow prereplicative complex assembly, no DNA replication initiates when a plasmid is directly incubated in NPE (Walter et al. 1998). Therefore, chromatin assembly in this experiment was mediated mostly by the HIRA-dependent pathway (see Supplemental Fig. S5A,B).

A single-strand break or DNA-bound PCNA can induce strand-specific MMR in vitro (Holmes et al. 1990; Thomas et al. 1991; Pluciennik et al. 2010; Kawasoe et al. 2016). If neither feature is present, MutSα or MutSβ binds to mismatches and translocates along DNA as a sliding clamp, but MutLα-dependent strand incision does not occur. Since the signal search reaction should require remote communication between a mismatch and strand signal, we wondered whether this step is associated with any alterations in chromatin structure. Interestingly, we found that a plasmid carrying an A:C mismatch (pMM1^AC^) (Fig. 1A) is not significantly supercoiled in NPE (Fig. 1B). Other base–base mismatches and a single IDL also inhibited supercoiling of a plasmid (Supplemental Fig. S1A). To see whether the inhibition of supercoiling is due to a reduction of the number of nucleosomes on DNA, we digested the plasmid with micrococcal nuclease (MNase). Southern blotting of the DNA showed that the mismatch-proximal region was highly sensitive to MNase (Fig. 1C). To quantitatively map the region where the MNase sensitivity is increased, we repeated the MNase digestion assay in the presence of an unrelated “control” plasmid (pControl) and quantified undigested DNA fragments by quantitative PCR (qPCR). This assay confirmed that the mismatch-proximal region (P1) is highly susceptible to MNase, and even the most mismatch-distal region (P5), which is ∼1.5 kb away from the mismatch, is weakly affected (Fig. 1D; Supplemental Fig. S1B). The MNase sensitivity of the control plasmid was not detectably changed by the coincubation of pMM1^AC^ (Fig. 1D, “pControl”), indicating that the increase of the MNase sensitivity occurs in *cis*. We also found that the relative MNase sensitivity is most strongly enhanced within an ∼1-kb region surrounding the mismatch (Fig. 1E,F; Supplemental Fig. S1C–E). Here, we refer to this reaction as nucleosome exclusion.

### Nucleosome exclusion depends on the Msh2-containing complexes and involves nucleosome disassembly

**Figure 2.**
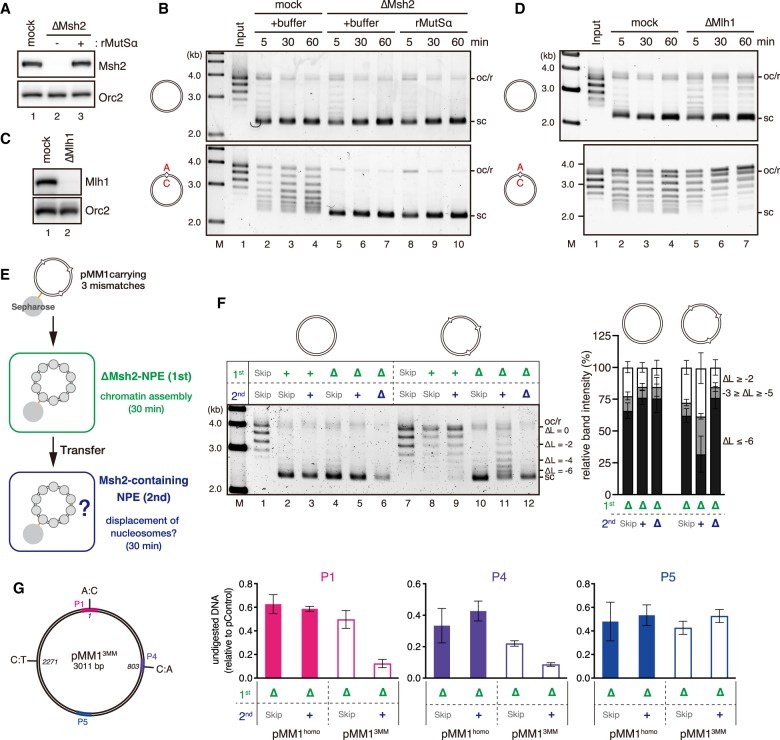
Nucleosome exclusion depends on the Msh2-containing complexes and involves nucleosome disassembly. (*A*) The immunodepletion efficiency of Msh2. To enhance the depletion efficiency, a mixture of Msh2 and Msh6 antibodies was used. Mock-treated (lane *1*, mock) or Msh2-depleted (and Msh6-depleted) NPE (lanes *2*,*3*, ΔMsh2) (0.25 µL of each) supplemented with either buffer (lanes *1*,*2*) or 900 nM recombinant MutSα (lane *3*) was separated by SDS-PAGE and probed with the indicated antibodies. Orc2 served as a loading control. The depletion efficiency was estimated as 99%. See also Figure 4A for codepletion of Msh6, Msh3, and other factors. (*B*) Supercoiling assay with pMM1^homo^ (*top*) or pMM1^AC^ (*bottom*) in NPE as described in *A*. See Supplemental Figure S2A–D for supercoiling and gap-directed MMR in Msh2- or Msh6-depleted NPE. (*C*) The immunodepletion efficiency of Mlh1. The depletion efficiency was estimated as 98%. (*D*) Supercoiling assay in NPE as described in *C*. See Supplemental Figure S2H for gap-directed MMR. (*E*) Schematic diagram of the nucleosome displacement assay. pMM1 carrying three mismatches at positions 1 (A:C), 803 (A:C), and 2271 (T:C) (pMM1^3MM^; see also *G*) was immobilized on Sepharose beads and incubated in an Msh2-depleted NPE for 30 min. The plasmid was then transferred into the second NPE containing Msh2, incubated for an additional 30 min, and recovered. (*F*) Nucleosome displacement assay. pMM1^homo^ (lanes *1*–*6*) or pMM1^3MM^ (lanes *7*–*12*) was sequentially incubated in the indicated extracts. (+) Mock-treated NPE; (Δ) Msh2-depleted NPE; (Skip) no incubation. The linking number of each band relative to the open circular or relaxed DNA (oc/r) position (ΔL) is indicated at the *right* of the gel. The ratio of the plasmids of the indicated ΔL was quantified and is presented as a graph. Mean ± one SD is shown. *n* = 5. (*G*) The nucleosome displacement assay was repeated without plasmid immobilization and in the presence of pControl. Instead of transferring plasmids, the second NPE was added directly to the first NPE to supply Msh2. The amount of DNA fragments relative to pControl after 60 sec of MNase digestion was quantified by qPCR. Mean ± one SD is shown. *n* = 3.

To test whether nucleosome exclusion depends on the Msh2-dependent MMR system, we immunodepleted both MutSα and MutSβ from NPE (Fig. 2A). As shown in Figure 2B, depletion of the Msh2-containing complexes relieved the inhibition of supercoiling on the mismatch-carrying plasmid. Depletion of Msh6 was sufficient to both allow supercoiling of the mismatch-carrying plasmid and inhibit gap-directed MMR of a base–base mismatch (Supplemental Fig. S2A–D). Depletion of NPE with four different Msh2 or Msh6 antibodies consistently allowed supercoiling of the mismatch-carrying plasmid (Supplemental Fig. S2A,B), strongly suggesting that the MutSα complex is primarily responsible for nucleosome exclusion around a base–base mismatch. However, the mismatch-dependent inhibition of supercoiling was not efficiently restored by the addition of recombinant MutSα to Msh2-depleted NPE (Fig. 2B). The reason for the failure of the rescue is currently not clear, but unidentified factors required for nucleosome exclusion might be codepleted with MutSα. It should also be noted that recombinant MutSα can restore gap-directed MMR in NPE (Kawasoe et al. 2016), suggesting that the level of nucleosome exclusion that is detectable in the supercoiling assay is dispensable for the repair of mismatches at least in the gap-directed system with naked DNA substrates (see below). Msh3 is >100-fold less concentrated than Msh2 in *Xenopus* egg extracts (Supplemental Fig. S2E), and, possibly because of its low concentration, the effect of MutSβ depletion on nucleosome exclusion was not visible in our experiments (Supplemental Fig. S2F,G). Depletion of Mlh1, which abolished gap-directed MMR (Supplemental Fig. S2H), did not inhibit nucleosome exclusion (Fig. 2C,D), suggesting that the Mlh1-containing complexes (Mlh1–Pms2, Mlh1–Pms1, and Mlh1–Mlh3) are dispensable for nucleosome exclusion. From these data, we infer that an Msh2-dependent but Mlh1-independent reaction facilitates nucleosome exclusion.

We also tested whether the exclusion reaction involves the displacement of preassembled nucleosomes (Fig. 2E). To enhance nucleosome exclusion, we used a 3.0-kb plasmid carrying three mismatches. The mismatch-carrying plasmid was fully supercoiled in an Msh2-depleted NPE (Fig. 2F, lane 10; see Supplemental Fig. S2I for Msh2 depletion). However, upon transfer into the second Msh2-containing NPE, plasmids with relative linking numbers of less than −6 were detectably decreased (Fig. 2F, cf. lanes 11 and 12). The displacement of nucleosomes likely occurred around the site of mismatches, since the MNase sensitivity was increased preferentially around mismatch sites (Fig. 2G). These data suggest that nucleosome exclusion is associated with active disassembly of nucleosomes.

### Chromatin remodeling enzyme Smarcad1 is recruited to mismatch-carrying DNA

**Figure 3.**
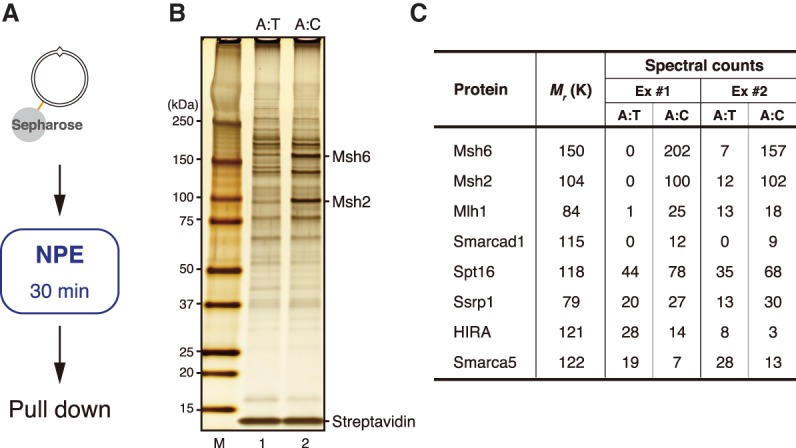
Identification of factors that are recruited onto mismatch-carrying DNA. (*A*) Schematic diagram of the plasmid pull-down assay. (*B*) Silver staining of mismatch DNA-binding factors. Samples were separated by SDS-PAGE and stained with silver nitrate. (A:T) pMM1^homo^; (A:C) pMM1^AC^. “M” indicates size markers. (*C*) Summary of mass spectrometry analysis. Spectral counts of the indicated proteins are listed along with their molecular masses. (Ex #1 and Ex #2) Independent experiments. See also Supplemental Table S1 for the complete list of identified factors.

To clarify the mechanism of nucleosome exclusion, we looked for factors that are recruited to mismatch-carrying DNA by Msh2-containing complexes. We immobilized plasmid DNA on Sepharose beads through a site-specific biotin modification (see Fig. 1A), incubated them in NPE, recovered the plasmid DNA, and compared the relative abundance of chromatin-binding factors by mass spectrometry (Fig. 3A–C). As expected, peptides corresponding to Msh2, Msh6, and Mlh1 were found preferentially on the mismatch DNA (Fig. 3C). The spectral counts of known chromatin-related factors such as HIRA and Smarca5 (ISWI) were reduced in the presence of a mismatch, probably because DNA was less chromatinized. However, the spectral counts of Smarcad1 and the FACT subunits Spt16 and Ssrp1 were increased in the presence of a mismatch.

**Figure 4.**
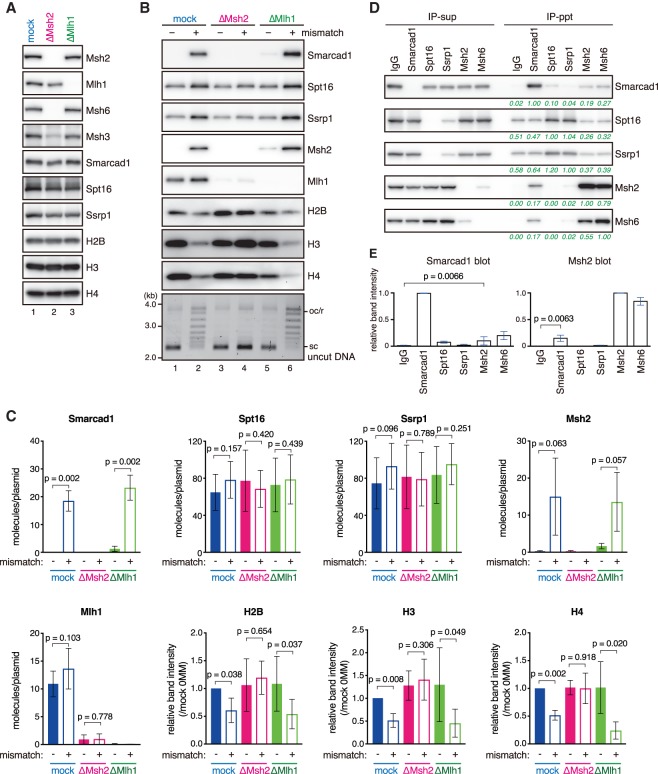
Smarcad1 is recruited onto mismatch-carrying DNA in a Msh2-dependent manner. (*A*) The immunodepletion efficiencies of Msh2 or Mlh1. NPE was depleted using preimmune antibodies (lane *1*, mock), a mixture of Msh2 and Msh6 antibodies (lane *2*, ΔMsh2), or Mlh1 antibodies (lane *3*, ΔMlh1). NPE (0.25 µL) was separated by SDS-PAGE and probed with the indicated antibodies. The depletion efficiencies for Msh2 and Mlh1 were estimated as 99% and 98%, respectively. (*B*) Immobilized pMM1^homo^ (lanes *1*,*3*,*5*) or pMM1^AC^ (lanes *2*,*4*,*6*) was incubated in NPE as described in *A* and recovered. Immunoblotting of the indicated antibodies and uncut DNA stained with SYBR Gold is presented. (*C*) Quantification of chromatin-binding factors. Band intensities were normalized to the amount of DNA quantified by qPCR. For Smarcad1, Msh2, Mlh1, Spt16, and Ssrp1, the number of molecules on a plasmid was estimated by using recombinant proteins as standards. Histones were normalized to the amount on no mismatch DNA in the mock sample. Mean ± one SD is shown. *n* = 4. *P*-values were calculated by the paired *t*-test (two-tailed). (*D*) Coimmunoprecipitation of Smarcad1 and MutSα. Immunoblotting of supernatant (IP-sup) and bead (IP-ppt) samples is presented. Green numbers represent band intensities relative to the target protein of immunoprecipitation. (*E*) Quantification of immunoprecipitated proteins. Mean ± one SD is shown. *n* = 3. *P*-values were calculated by the paired *t*-test (two-tailed).

To quantitatively compare chromatin binding of these factors, we repeated the plasmid pull-down assay and blotted each factor with specific antibodies (Fig. 4A–C; Supplemental Fig. S3). Loading of Histones H2B, H3, and H4 was significantly reduced in the presence of a mismatch, and this effect was dependent on Msh2 but not Mlh1 (Fig. 4A–C). Smarcad1 was specifically recruited onto the mismatch-carrying DNA (Fig. 4B [lanes 1,2], C). Critically, mismatch-specific loading of Smarcad1 was dependent on Msh2 but not Mlh1 (Fig. 4B [lanes 2,4,6], C). Consistent with the mass spectrometry data, Spt16 and Ssrp1 were found on DNA in the absence of a mismatch. Chromatin binding of FACT subunits may be increased in the presence of a mismatch, but the difference was not statistically significant with our sample number (*n* = 4) (Fig. 4C). By immunoprecipitation, a small amount of Smarcad1 was coprecipitated with Msh2 and Msh6, and Smarcad1 coprecipitated Msh2 and Msh6 (Fig. 4D,E), suggesting that Smarcad1 physically interacts with MutSα, as reported in human cells (Okazaki et al. 2008; Rowbotham et al. 2011; Chen et al. 2016). These results identify Smarcad1 as a factor that is recruited onto mismatch-carrying DNA by an Msh2-dependent mechanism. Since FACT showed very high nonspecific binding to control IgG beads, we were not able to perform reliable experiments on the interaction between FACT and MutSα (Fig. 4D).

### Smarcad1 promotes mismatch-dependent exclusion of nucleosomes

**Figure 5.**
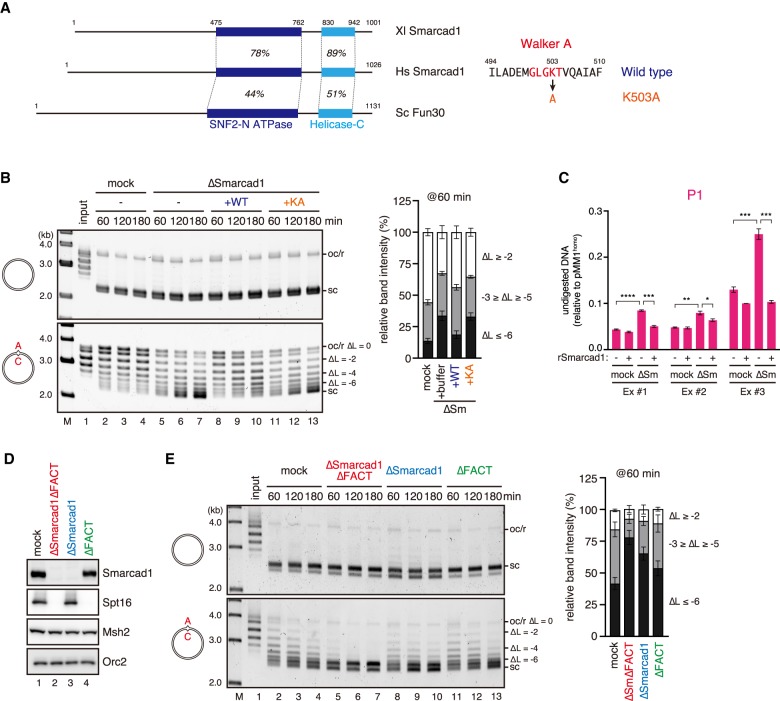
Smarcad1 and FACT assist nucleosome exclusion. (*A*) The domain architecture of *Xenopus laevis* (Xl) and *Homo sapiens* (Hs) Smarcad1 and *Saccharomyces cerevisiae* (Sc) Fun30. The positions and sequence identities of the SNF2 family N-terminal domain (SNF2-N ATPase) and helicase C-terminal domain (Helicase-C) are indicated. The sequence of isoform A was used for Xl Smarcad1. The amino acid sequence of the Walker A motif is presented. (*B*) Supercoiling assay in Smarcad1-depleted NPE. The linking number of each band relative to the open circular or relaxed DNA (oc/r) position (ΔL) is indicated. The ratio of the plasmids of the indicated ΔL was quantified and is presented as a graph. Mean ± one SD is shown. *n* = 3. See Supplemental Figure S4A for depletion efficiencies. (*C*) The MNase assay was performed as described in Figure 1D using Smarcad1-depleted NPE. (Ex #1, Ex #2, and Ex #3) Independent experiments. The amount of undigested DNA relative to pMM1^homo^ is plotted as a graph. Mean ± one SD is shown. *n* = 3 technical replicates. *P*-values were calculated by the unpaired *t*-test (two-tailed). (*) *P* < 0.05; (**) *P* < 0.01; (***) *P* < 0.001; (****) *P* < 0.0001. (*D*) Immunodepletion efficiencies of Smarcad1 and FACT. Mock-treated (lane *1*), FACT- and Smarcad1-depleted (lane *2*), Smarcad1-depleted (lane *3*), or FACT-depleted (lane *4*) NPE was separated by SDS-PAGE and probed with the indicated antibodies. NPE (0.25 µL of each) was loaded. The depletion efficiencies for Smarcad1 and Spt16 were estimated as 98% and 95%, respectively. (*E*) The supercoiling assay in NPE described in *D*. The ratio of the plasmids of the indicated ΔL was quantified and is presented as a graph. Mean ± one SD is shown. *n* = 3.

We tested whether Smarcad1 promotes nucleosome exclusion. In an NPE depleted of ∼98% Smarcad1, we did not see a detectable change in supercoiling in the absence of a mismatch, suggesting that Smarcad1 does not play a major role in nucleosome assembly in this system (Fig. 5A,B; see Supplemental Fig. S4A for Smarcad1 depletion). In the presence of a mismatch, however, plasmids that have relative linking numbers of less than −3 were accumulated, and this accumulation was reverted by the addition of wild-type but not the Walker A mutant Smarcad1 (Fig. 5B; Supplemental Fig. S4F,G [see B for recombinant Smarcad1]). We also found that regions surrounding the mismatch become more resistant to MNase in the absence of Smarcad1, an effect that is reversed by recombinant Smarcad1 (Fig. 5C; Supplemental Fig. S4C–E). These results suggest that Smarcad1 functions as an ATPase to facilitate nucleosome exclusion.

As the effect of Smarcad1 depletion was partial compared with Msh2 depletion, we included FACT in our supercoiling assay to see whether FACT also contributes to nucleosome exclusion (Fig. 5D,E). Although depletion of FACT by itself had no detectable effect, simultaneous depletion of Smarcad1 and FACT further enhanced supercoiling of the plasmid in the presence of a mismatch (Fig. 5D,E; Supplemental Fig. S4H,I). These data suggest that FACT also assists nucleosome exclusion, albeit to a lesser extent.

### Nucleosome exclusion counteracts synthesis-coupled chromatin assembly

**Figure 6.**
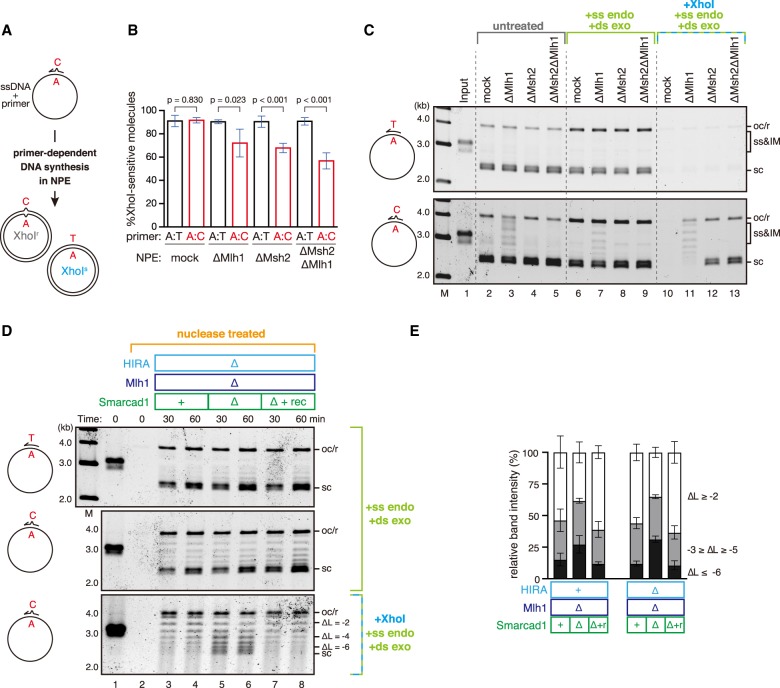
The nucleosome exclusion reaction counteracts DNA synthesis-coupled chromatin assembly. (*A*) Schematic diagram of the primer extension assay. A 92-nucleotide (nt) primer carrying either no mismatch or an A:C mismatch is annealed on a single-stranded pMM1. Upon incubation in NPE, complementary DNA is synthesized depending on the primer, converting the substrate into covalently closed circular DNA. (*B*) The requirements of canonical MMR factors for primer extension-coupled mismatch correction. The primer extension assay was performed in mock-treated, Mlh1-depleted (ΔMlh1), Msh2-depleted (ΔMsh2), or Msh2/Mlh1 doubly depleted (ΔMsh2ΔMlh1) NPE. The ratio of XhoI-sensitive molecules that correspond to the C-to-T repair products is plotted in a graph. Mean ± one SD is shown. *n* = 4. *P*-values were calculated by the unpaired *t*-test (two-tailed). Note that the ratio did not reach 100% even with a homoduplex primer because of the presence of some residual primer extension intermediates. See also Supplemental Figure S6, D and E, for the details of quantification. (*C*) Nucleosome exclusion on the primer extension products. The products described in *B* were separated by agarose gel without any treatment (lanes *2*–*5*), after digestion of incomplete intermediates by S1 nuclease and ExoV (lanes *6*–*9*), or after digestion of C-to-T repair products and incomplete intermediates by XhoI, S1 nuclease, and λ exonuclease (lanes *10*–*13*). (ss) ssDNA; (IM) primer extension intermediates. (*D*) The assay presented in *C* was repeated in NPE depleted of Mlh1 and HIRA (lanes *3*,*4*) or Mlh1, HIRA, and Smarcad1 (lanes *5*–*8*) supplemented with either buffer (lanes *3*–*6*) or recombinant Smarcad1 (lanes *7*,*8*). The linking number of each band relative to the open circular or relaxed DNA (oc/r) position (ΔL) is indicated at the *right* of the gel. (*E*) The ratio of the plasmids of the indicated ΔL in *D* and Supplemental Figure S5F was quantified and is presented as a graph. Mean ± one SD is shown. *n* = 3.

Since MMR is a post-replicative repair system, CAF-1-mediated chromatin assembly may be more relevant to MMR. NPE efficiently converts a primed single-stranded plasmid to the double-stranded form (Fig. 6A). Because unregulated priming is suppressed in NPE (Walter and Newport 2000), DNA synthesis initiates from the 3′ terminus of the primer, and the MMR system can use either terminus of the primer as a strand discrimination signal. In this system, as expected, supercoiling of the primer extension products depended on both HIRA and CAF-1, while that of double-stranded plasmids depended only on HIRA (Supplemental Fig. S5A,B). A mismatch on the primer was efficiently repaired, and the repair was partially dependent on Msh2 and Mlh1 (Fig. 6B; Supplemental Fig. S5C). The mismatch correction seen in Msh2- and Mlh1-depleted NPE may be mediated by proofreading by DNA polymerases or flap processing during the completion of synthesis. Importantly, in the absence of Mlh1, DNA products that escaped from Mlh1-independent mismatch correction were not supercoiled (Fig. 6C, lane 11; Supplemental Fig. S5D), and the inhibition of supercoiling was Msh2-dependent (Fig. 6C, lane 13). Since depletion of either HIRA or CAF-1 was insufficient for preventing supercoiling of the primer extension products, we infer that nucleosome exclusion can counteract both HIRA- and CAF-1-mediated chromatin assembly.

To test how Smarcad1 contributes to the inhibition of supercoiling in this system, we combined depletion of Smarcad1 with that of Mlh1 and HIRA. Indeed, in the absence of Smarcad1, regardless of the presence or absence of HIRA, the plasmids with relative linking numbers of less than −6 were significantly accumulated, and the effect was reversed by recombinant Smarcad1 (Fig. 6D,E; Supplemental Fig. S5E–H). These data are in good agreement with the hypothesis that Smarcad1 assists Msh2-dependent nucleosome exclusion to counteract both HIRA- and CAF-1-mediated chromatin assembly.

### Smarcad1 facilitates the repair of mismatches when DNA is chromatinized

**Figure 7.**
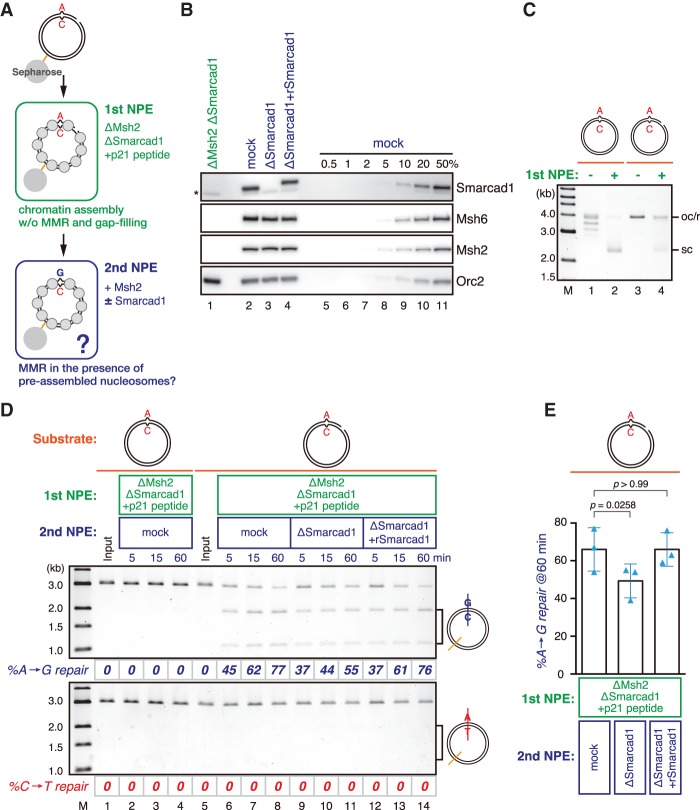
Smarcad1 facilitates MMR in the presence of preassembled nucleosomes. (*A*) Schematic diagram of the stepwise incubation assay. pMM1^AC^ carrying a 15-nt gap on the A strand was immobilized on Sepharose beads and incubated in an Msh2- and Smarcad1-depleted NPE (1st NPE) containing 1 mg/mL p21 PCNA-binding peptide (NH_2_-KRRQTSMTDFYHSKRRLIFS-COOH) for 30 min. The plasmid was then transferred into the second NPE (2nd NPE) containing Msh2 and incubated for the indicated times. (*B*) The immunodepletion efficiencies of MutSα and Smarcad1. (*) Cross-reacting band. (*C*) Supercoiling assay in the first NPE. See also Supplemental Figure S6, F and G, for the effect of the p21 peptide on gap retention. (*D*) MMR efficiencies after the incubation in the second NPE. DNA was digested with XmnI and either BamHI (A-to-G repair; *top*) or XhoI (C-to-T repair; *bottom*). The percentage of repair was calculated based on the percentage of XhoI- or BamHI-sensitive DNA molecules. (*E*) Statistical analysis of the effect of Smarcad1 on MMR in the stepwise incubation assay. The A-to-G repair efficiencies are plotted in a graph. Mean ± one SD is shown. *n* = 3. *P*-values were calculated by the paired *t*-test (two-tailed). Blue triangles indicate individual values.

An important question is whether Smarcad1 and FACT promote replication error correction in the context of chromatin. Because it is not possible at this point to quantitatively measure replication-coupled error correction in *Xenopus* egg extracts, we undertook three approaches. First, we used the gap-directed MMR assay. However, we observed no reproducible reduction in the MMR efficiencies by depletion of Smarcad1, FACT, or both even when the mismatch gap distance was extended to 1.9 kb (Supplemental Fig. S6A,B). Second, we used the primer extension-based MMR assay. Again, we did not see a detectable reduction of the MMR efficiencies by depletion of Smarcad1 (Supplemental Fig. S6C–E). In both approaches, we started the reaction by adding naked DNA substrates into NPE. If the recognition of mismatches is significantly quicker than chromatin assembly, these approaches may not be appropriate for testing the effect of chromatin on MMR. Third, we set up a system where MMR is initiated on a chromatinized template. We preassembled nucleosomes on a gap-carrying DNA in the absence of Msh2 and Smarcad1 and then supplied Msh2 to initiate the MMR reaction (Fig. 7A). Since the strand discrimination signal (a gap) is quickly filled in NPE, we repressed gap filling by inhibiting the PCNA function with a PCNA-binding peptide derived from p21 (Supplemental Fig. S6F,G; Kawasoe et al. 2016). Stepwise incubation did not significantly reduce the MMR efficiency when the gap was retained by the p21 peptide (Supplemental Fig. S6G, cf. lanes 1–4 and 13–16). Interestingly, however, we observed a statistically significant reduction of the MMR efficiency in the absence of Smarcad1, and the effect was restored by recombinant Smarcad1 (Fig. 7B–E). These results suggest that Smarcad1 facilitates Msh2-dependent MMR when nucleosomes are assembled around a mismatch.

### The yeast homolog of Smarcad1 is an accessory factor for Msh2-dependent MMR

To assess the contribution of the Smarcad1 homolog to replication error correction in the context of chromatin replication, we used the budding yeast *Saccharomyces cerevisiae*. Homopolymer runs of adenine or thymine are known hot spots for slippage of DNA polymerases, and MMR very efficiently corrects such slippage errors (Kunkel and Erie 2015). In this study, we measured the reversion of *hom3-10* and *lys2::insE-A14*, both of which detect −1 frameshifts in A/T runs (Marsischky et al. 1996; Tran et al. 1997).

The budding yeast genome encodes one Smarcad1 homolog, Fun30 (see Fig. 5A). *fun30Δ* increased the reversion rate by 2.1-fold (*P*-value = 0.0024, wild type vs. *fun30Δ*) at *hom3* and by 1.9-fold (*P*-value < 0.0001) at *lys2*, suggesting a possibility that Fun30 contributes to the suppression of spontaneous mutations (Table 1). To test the possibility that Fun30 is an accessory factor for the MMR system, we evaluated genetic interactions between Fun30 and MMR factors by making double mutants. In budding yeast, due to their functional overlap, either *msh6Δ* (ΔMutSα) or *msh3Δ* (ΔMutSβ) causes only a partial increase of the mutation rates for these frameshift assays (Marsischky et al. 1996). Interestingly, when *FUN30* was disrupted simultaneously with *MSH6* or *MSH3*, the reversion rates increased synergistically rather than additively. For instance, *fun30Δ* increased the reversion rate by ∼12-fold in the *msh6Δ* background at *hom3* and approximately sixfold at *lys2*. *fun30Δ* increased the reversion rates by approximately twofold in *msh3Δ* cells at both loci, and this increase was much higher than the sum of the rates from every single mutant. Importantly, *fun30Δ* did not increase the reversion rates in *msh2Δ* cells. This is consistent with the idea that *FUN30* contributes to a mutation avoidance pathway epistatic to *MSH2*. The phenotype of the Walker A mutant of Fun30 (*fun30-K603A*) closely resembled that of *fun30Δ*, suggesting that an ATP-dependent function is required for Fun30 to suppress mutations. To test whether the genetic interaction is specific to Msh2-containing complexes, we combined *fun30Δ* with *exo1Δ*, which also partially impairs MMR (Tishkoff et al. 1997; Amin et al. 2001). Critically, *fun30Δ* did not show a synergistic increase in the reversion rates with *exo1Δ*, and the genetic interaction between *FUN30* and *MSH6* was kept in the *exo1Δ* cells (cf. *exo1Δ msh6Δ* and *exo1Δ msh6Δ fun30Δ*). These factor-specific genetic interactions suggest that the function of Fun30 in MMR is related to MutSα and MutSβ. As MutSα suppresses recombination between divergent sequences and since Fun30 is involved in the repair of DSBs (Chen et al. 2012; Costelloe et al. 2012; Eapen et al. 2012; Densham et al. 2016), we tested whether the observed genetic interactions are dependent on recombination (Supplemental Table S2). However, the synergistic effects were still observed in the homologous recombination-deficient *rad52Δ* background (cf. *rad52Δ msh6Δ* and *rad52Δ msh6Δ fun30Δ*), and the frameshift mutations seen in *fun30Δ* cells were more concentrated in the homopolymer “hot spot” runs, as seen in MMR mutants, than in *rad52Δ* cells (Supplemental Fig. S7A,B). These data collectively suggest that an ATP-dependent function of Fun30 cooperates with the Msh2-containing complexes (especially with MutSβ, at least for these genetic systems) to facilitate replication error correction. A temperature-sensitive mutant of FACT, *spt16-d922* (Evans et al. 1998), did not show a significant mutator phenotype even when combined with *msh6Δ* (Supplemental Table S3). *spt16-d922* did not elevate the reversion rates also when combined with *fun30Δ*. As FACT is essential, we were not able to test the effect of *factΔ*, leaving open the question of whether FACT facilitates MMR in yeast. Given the functional overlap between the two factors in *Xenopus*, it is still possible that yeast FACT has a role redundant with Fun30 in MMR.

**Table 1.**
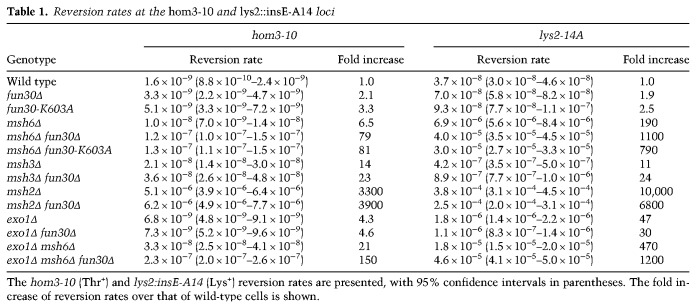
Reversion rates at the *hom3-10* and *lys2::insE-A14* loci

### Fun30 counteracts CAF-1 to assist Msh2-dependent MMR

If Fun30 cooperates with the Msh2-containing complexes to counteract chromatin assembly, impairment of chromatin assembly should mitigate the mutator phenotype of *fun30Δ*. To test this possibility, we deleted *CAC1*, the largest subunit of CAF-1. *cac1Δ* alone slightly decreased the mutation rate at *hom3* and slightly increased the rate at *lys2* (Table 2). The difference may be related to specific sequences or chromatin structure at two loci. Interestingly, however, in *msh6Δ fun30Δ* cells, *cac1Δ* decreased the reversion rates by more than fivefold at *hom3* and threefold at *lys2*. The reduction of reversion rates suggests that the majority of mutations seen in the *msh6Δ fun30Δ* double mutant is caused through CAF-1 function. *cac1Δ* also reduced mutation rates in not only *msh3Δ fun30Δ* cells but also *msh6Δ* or *msh3Δ* cells, suggesting that CAF-1 is inhibitory for both MutSα- and MutSβ-dependent MMR. Importantly, *cac1Δ* did not significantly change the reversion rates in *msh2Δ* cells, implying that the effect of *cac1Δ* is epistatic to *msh2Δ*. From these results, we infer that CAF-1 impedes Msh2-dependent MMR, and Fun30, MutSα, and MutSβ counteract the function of CAF-1 to facilitate replication error correction.

**Table 2.**
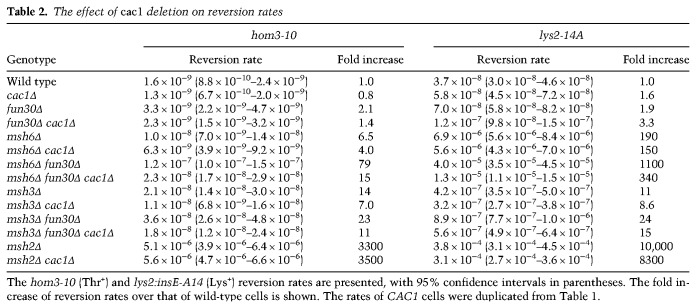
The effect of *cac1* deletion on reversion rates

## Discussion

How the MMR machinery handles nucleosomes around mismatched bases is a key question for understanding the MMR reaction in the context of chromatin. Previous studies have shown that eukaryotic cells have multiple mechanisms to function on chromatinized DNA, which is shown to be inhibitory for MMR in vitro (Li et al. 2009; Gorman et al. 2010; Schopf et al. 2012; Brown et al. 2016) but may not be so in vivo (Lujan et al. 2014). MutSα has a chromatin remodeling activity and interferes with CAF-1 function, likely to promote MMR on chromatin (Javaid et al. 2009; Kadyrova et al. 2011; Schopf et al. 2012; Rodriges Blanko et al. 2016). It is also possible that nucleosomes and other obstructions on DNA are not inhibitory for the MutLα step in MMR, as eukaryotic MutLα and bacterial MutL form a large proteinaceous ring that can bypass nucleosomes or MutS while traveling along DNA (Gorman et al. 2010; Liu et al. 2016). In this study, using *Xenopus* egg extracts that recapitulate chromatin assembly and MMR in vitro, we discovered that the eukaryotic MMR system has a remarkable ability to exclude nucleosomes around a mismatched base. We further showed that the SNF2 family chromatin remodeler Smarcad1 assists both nucleosome exclusion and the repair of mismatches on chromatinized DNA. Genetic experiments in yeast provided evidence that the yeast homolog of Smarcad1 contributes to the MutSα- and MutSβ-dependent MMR by counteracting the function of CAF-1. Both the biochemical and genetic data indicate that Smarcad1/Fun30 is an MMR accessory factor that assists the function of Msh2-containing mismatch sensor complexes.

Our data showed that nucleosome exclusion occurs at the step of Msh2-dependent mismatch recognition. Thus, Msh2, but not Mlh1, is required for exclusion of nucleosomes, and nucleosome exclusion occurs in the absence of strand discrimination signals. Consistent with this, chromatin loading of Smarcad1 was dependent on Msh2 but not on Mlh1. It is noteworthy that we observed no detectable loading of Smarcad1 in our plasmid pull-down assay in the absence of a mismatch. This finding suggests that Smarcad1 is recruited to chromatin through the interaction with MMR proteins rather than with nucleosomes. Previous reports have also shown that the Smarcad1 homologs function at rather specific chromosomal loci such as the site of DSBs, heterochromatin, and centromeres (Neves-Costa et al. 2009; Stralfors et al. 2011; Chen et al. 2012; Costelloe et al. 2012; Eapen et al. 2012; Densham et al. 2016; Taneja et al. 2017). Although only a small portion of Smarcad1 was pulled down with Msh2 and Msh6 by immunoprecipitation (Fig. 4D), the amount of Smarcad1 on a mismatch-carrying DNA was comparable with that of Msh2 (Fig. 4C). As the MutSα and MutSβ complexes change their conformation upon the recognition of a mismatch, it would be possible that the ATP-bound form of MutSα—and also possibly MutSβ—preferentially interacts with Smarcad1 to load it onto DNA. We also showed that simultaneous depletion of Smarcad1 and FACT further weakens nucleosome exclusion. Interestingly, FACT is a histone chaperone that promotes histone exchange (Formosa 2012), and yeast Fun30 also shows a histone exchange activity in vitro (Awad et al. 2010). Smarcad1 and FACT might assist nucleosome exclusion by accelerating the turnover rate of histones. More mechanistic analyses are needed to clarify how these factors contribute to the exclusion of nucleosomes around a mismatch.

An important question is how nucleosome exclusion and Smarcad1 contribute to post-replicative MMR. Our stepwise incubation experiments showed that, at least in a situation where nucleosomes are assembled around a mismatch before the initiation of MMR, Smarcad1 contributes to the repair of the mismatch (Fig. 7). This finding supports a hypothesis that Smarcad1 collaborates with Msh2-containing complexes to promote the repair of mismatches by displacing local nucleosomes. However, at this point, it is difficult to estimate the contribution of nucleosome exclusion to in vivo MMR. Our biochemical data indicate that Smarcad1's contribution to nucleosome exclusion is partial, and, if this is also true in yeast, the mutator phenotype of *fun30Δ* may represent only a part of the contribution of nucleosome exclusion to MMR. Interestingly, in our experiments, recombinant MutSα restores gap-directed MMR (Kawasoe et al. 2016) but not nucleosome exclusion in Msh2-depleted NPE (Fig. 2B). This finding suggests that Msh2 depletion likely codepletes a factor that is critical for nucleosome exclusion. Since Smarcad1 is only partially required for nucleosome exclusion and since Smarcad1 is not quantitatively codepleted with Msh2, the factor that is codepleted with Msh2 would not be Smarcad1. Identification of such factors may clarify the relative contribution of nucleosome exclusion to MMR. Currently, however, it is still possible that our recombinant MutSα lacks some specific activity that is critical for nucleosome exclusion.

The genetic data in yeast were consistent with the biochemical data and at least partially compensated for limitations of the in vitro experiments. Thus, *fun30Δ* exacerbated the reversion rates in *msh6Δ* and *msh3Δ* cells but not in *msh2Δ* cells, suggesting that Fun30 cooperates with the Msh2-containing complexes to assist replication error correction. In addition, deletion of *CAC1* reduced the reversion rates in *fun30Δ msh6Δ* cells, suggesting that Fun30 has a role in counteracting CAF-1. This is in a good agreement with biochemical data in *Xenopus*; Smarcad1 facilitates mismatch correction on a chromatinized template (Fig. 7) and assists nucleosome exclusion by counteracting CAF-1-mediated chromatin assembly (Fig. 6). It should also be noted that our genetic experiments are in good agreement with the data showing that CAF-1 is inhibitory for MMR (Kadyrova et al. 2011; Schopf et al. 2012; Rodriges Blanko et al. 2016) and suppresses the cytotoxic activity of the MMR system upon treatment with a DNA-alkylating agent (Kadyrova et al. 2016). Curiously, the genetic data suggest that yeast Fun30 is more important for MutSβ-dependent MMR than for MutSα-dependent MMR. Because MutSα has an ability to counteract CAF-1-mediated chromatin assembly (Javaid et al. 2009; Kadyrova et al. 2011; Schopf et al. 2012; Rodriges Blanko et al. 2016), it could be less dependent on Fun30 than MutSβ, although it is not clear at this point whether MutSβ lacks such an ability. However, as we measured reversions at only two loci and detected exclusively −1 frameshifts, genome-wide evaluation of unbiased mutation rates and spectra in *fun30Δ* cells would be essential for a comprehensive understanding of the Fun30 function in MMR. Likewise, although the genetic interaction between *FUN30*, MMR factors, and *CAC1* is generally in good agreement with a scenario in which Fun30 counteracts CAF-1-mediated chromatin assembly to assist MMR, other possibilities are currently not excluded. Establishment of a biochemical assay with yeast proteins will be a key to connecting the biochemical data in *Xenopus* and the genetic data in yeast.

In conclusion, our study established that the Msh2-dependent MMR system has an ability to exclude nucleosomes around mismatches and identified Smarcad1/Fun30 as an accessory factor for nucleosome exclusion and Msh2-dependent mismatch correction. Although post-replicative MMR may occur mostly just behind the replication fork, it is possible that nucleosome exclusion becomes more important for MMR after the completion of chromatin assembly. In the leading strand, ribonucleotides embedded by polymerase ε serve as strand discrimination signals after their conversion into single-strand gaps by ribonucleotide excision repair (Ghodgaonkar et al. 2013; Lujan et al. 2013). Since ribonucleotide excision repair occurs after nascent DNA synthesis, ribonucleotide-induced MMR is more likely to conflict with nucleosomes and could be more dependent on nucleosome exclusion. Nucleosome exclusion may also be involved in other Msh2-related reactions such as heteroduplex rejection during recombination, apoptosis upon alkylating DNA damage, and somatic hypermutation in immune cells. Our study serves as a basis for future investigations into these interesting possibilities.

## Materials and methods

### *Xenopus* egg extract

The NPE of *Xenopus* eggs was prepared essentially as described previously (Lebofsky et al. 2009). See the Supplemental Material for detail. *Xenopus laevis* was purchased from Kato-S-kagaku and maintained and handled according to the animal care regulations at Osaka University and Kyushu University.

### Yeast genetic analysis

All *S. cerevisiae* strains used in this study were derived from BY4741 and are listed in Supplemental Table S4. Mutation rates were estimated by fluctuation analysis using the Ma-Sandri-Sarkar (MSS) maximum likelihood method (Sarkar et al. 1992; Rosche and Foster 2000). Ninety-five percent confidence intervals were estimated based on the mutation rates obtained by the MSS method. For each replicate in the fluctuation analysis, a yeast culture was started from a single colony and grown to the stationary phase in 10 mL of yeast extract–peptone–dextrose medium plus adenine. Appropriate aliquots of cells were plated onto synthetic dextrose (SD) medium with amino acids lacking lysine or threonine to count Lys^+^ or Thr^+^ revertants and onto synthetic complete (SC) medium to count viable cells. For strains with very high mutation rates (strains carrying *msh2Δ*, *msh6Δ*, or *exo1Δ*), a single colony was directly suspended in 1 mL of distilled water, and appropriate aliquots were plated on solid medium. See the Supplemental Material for detail of the statistical testing.

### Supercoiling, gap-directed MMR, and primer extension assays

The supercoiling assay was carried out essentially as described previously (Kawasoe et al. 2016). NPE was supplemented with 2 mM ATP, 20 mM phosphocreatine (PC), and 5 µg/mL creatine phosphokinase (CPK) and preincubated for 5 min at 22°C. A typical reaction consisted of 17.4 µL of NPE, 0.2 µL of 200 mM ATP, 0.4 µL of 1 M PC, 0.02 µL of 5 mg/mL CPK, and 2 µL of substrate DNA (200 ng/µL in TE: 10 mM Tris-HCl, 1 mM ethylenediaminetetraacetic acid [EDTA] at pH 7.4). Gap-carrying DNA was used at a final concentration of 20 ng/µL for the gap-directed MMR assay, and primed ssDNA was used at a final concentration of 10 ng/µL for the primer extension assay. After adding DNA, reaction mixtures were incubated at 22°C, and aliquots (1.5–3 µL for most experiments) were stopped by addition of 100 µL of 1% SDS in 20 mM EDTA. DNA was purified by proteinase K treatment, phenol/chloroform extraction, and ethanol precipitation. To analyze the MMR efficiency, 10 ng of DNA was digested with XmnI and BamHI-HF or XhoI (New England Biolabs) in a 10-µL reaction. In the primer extension assay, 30 ng of DNA was digested with 3 U of S1 nuclease (Takara) and 0.3 U of ExoV (New England Biolabs) or with 3 U of XhoI, 3 U of S1 nuclease, and 0.3 U of λ exonuclease (New England Biolabs) in 1× CutSmart buffer (New England Biolabs) in an 8-µL reaction. After agarose gel electrophoresis, DNA was stained with SYBR Gold nucleic acid stain (Life Technologies) and scanned with a Typhoon FLA9000 (GE Healthcare) or ChemiDoc Touch (Bio-Rad). Signal intensities were quantified using ImageJ (National Institutes of Health).

### MNase digestion, Southern blotting, and qPCR

A 17-µL supercoiling reaction was set up and incubated for 60 min at 22°C. A 2-µL aliquot was sampled for supercoiling, and another 15-µL aliquot was quickly diluted with 1.5 mL of MNase buffer (10 mM Tris-HCl, 50 mM NaCl, 2.5 mM CaCl_2_ at pH 7.4) containing 20 U/mL MNase (Worthington). The samples were incubated at 37°C; 350 µL each of aliquots was stopped by addition of 50 µL of C-stop buffer (160 mM EDTA, 6.8% SDS) at 15, 30, 60, and 120 sec; and DNA was purified. For Southern blotting, DNA was separated on 1.2% agarose gel in 0.5× TBE (Tris-borate-EDTA) buffer, stained with SYBR Gold, and scanned with a Typhoon FLA9000. DNA was then transferred onto Hybond N^+^ nylon membrane (GE Healthcare) and hybridized with a ^32^P-labeled probe prepared from the PvuII–PvuII 473-base-pair (bp) fragment of pMM1 using the random primer DNA labeling kit (Takara). The probe was stripped off after detection of ^32^P, and the membrane was rehybridized with another probe prepared from the DraI–DraI 692-bp fragment. β Rays from ^32^P were detected by a Typhoon FLA9000 using a phosphorimaging plate. For qPCR, supercoiling reactions were set up in the presence of 5 ng/µL pControl/pCDFDuet-1 (Merck Millipore). DNA samples were diluted in TE, and 10-µL reactions (7 µL of qPCR master mix, 2 µL of 1 µM primer mix, and 1 µL of diluted DNA) were run in a Mx3000P system (Stratagene) using KOD SYBR qPCR mix (Toyobo) and primers listed in Supplemental Table S5.

### Plasmid pull-down and mass spectrometry identification of DNA-bound proteins

Singly biotinylated plasmid DNA was immobilized on streptavidin-coated biotin-Sepharose beads as described previously (Kawasoe et al. 2016). Immobilized DNA was incubated in NPE at 20 ng/µL (600 ng of DNA bound to 6 µL of Sepharose in a 30-µL reaction) for 30 min at 22°C. The reaction mixture was diluted with 200 µL of 1× egg lysis buffer (ELB; 10 mM HEPES-KOH, 2.5 mM MgCl_2_, 50 mM KCl at pH 7.7) containing 0.2% Triton X-100, layered over 300 µL of ELB containing 500 mM sucrose, and centrifuged at 12,700*g* for 2 min at 4°C in a horizontal centrifuge (Tomy Seiko). The beads were washed three times with ELB, and bound proteins were eluted with 12 µL of Laemmli's SDS sample buffer (62.5 mM Tris-HCl, 10% glycerol, 3% SDS, 0.005% bromophenol blue, 5% 2-mercaptoethanol at pH 6.8). To monitor DNA recovery, DNA was extracted with phenol/chloroform, precipitated with ethanol, and dissolved in TE. The amount of DNA was determined by qPCR with primers 1842 and 1843. Mass spectrometry analysis was carried out as described previously with minor modifications (Nozawa et al. 2010). The LC-MS/MS data were searched against a *X. laevis* subset database created from RefSeq (release 82). Identified proteins were semiquantified by spectral counting (Liu et al. 2004) using Scaffold software version 4.8.3 (Proteome Software, Inc.).

### Stepwise incubation assay

Immobilized DNA was incubated in NPE as described in the method for plasmid pull-down. After a 30-min incubation, the DNA was recovered by centrifugation in a benchtop centrifuge, washed three times with ELB, and incubated in the second NPE at 20 ng/µL concentration (100 ng of DNA bound to 1 µL of Sepharose in a 5-µL reaction) for 30 min at 22°C unless stated otherwise. For the experiment shown in Figure 2G, biotin-free DNA was used as a substrate, and an equal volume of the second NPE was added directly to the reaction. The reaction was stopped by addition of 100 µL of 1% SDS in 20 mM EDTA. DNA was purified by proteinase K treatment, phenol/chloroform extraction, and ethanol precipitation.

## Supplementary Material

Supplemental Material
