# Supplementary material for: Nucleosomes around a mismatched base pair are excluded via an Msh2-dependent reaction with the aid of SNF2 family ATPase Smarcad1
Source: Genes Dev. 2018 Jun 1;32(11-12):806–21. doi: 10.1101/gad.310995.117 (PMC6049510; doi:10.1101/gad.310995.117)
Supplement: Supplemental Material [file supp_32_11-12_806__index.html]

Supplemental Material 

# Nucleosomes around a mismatched base pair are excluded via an Msh2-dependent reaction with the aid of SNF2 family ATPase Smarcad1

## Supplemental Material

- Supplemental\_information.pdf
- Supplemental\_table\_S1.xls
