# Supplementary material for: Nucleosomes around a mismatched base pair are excluded via an Msh2-dependent reaction with the aid of SNF2 family ATPase Smarcad1
Source: Genes Dev. 2018 Jun 1;32(11-12):806–21. doi: 10.1101/gad.310995.117 (PMC6049510; doi:10.1101/gad.310995.117)
Supplement: Supplemental Material [file supp_gad.310995.117_Supplemental_information_.pdf]

Nucleosomes around a mismatched base pair are excluded via an Msh2-dependent reaction with the aid of SNF2-family ATPase Smarcad1

Riki Terui, Koji Nagao, Yoshitaka Kawasoe, Kanae Taki, Torahiko L. Higashi, Seiji Tanaka, Takuro Nakagawa, Chikashi Obuse, Hisao Masukata, and Tatsuro S. Takahashi

Supplemental information

Supplemental Inventory:

Supplemental Materials and Methods

Supplemental References

Supplemental Figure Legends

Supplemental Figure S1 – related to Figure 1

Supplemental Figure S2 – related to Figure 2

Supplemental Figure S3 – related to Figure 4

Supplemental Figure S4 – related to Figure 5

Supplemental Figure S5 – related to Figure 6

Supplemental Figure S6 – related to Figure 7

Supplemental Figure S7 – related to Table 1

Supplemental Table S1 (separate excel file) – related to Figure 3

Supplemental Tables S2–5

Supplemental Materials and Methods

Preparation of the nucleoplasmic extract of *Xenopus* eggs (NPE)

Preparation of NPE was carried out essentially as described previously (Lebofsky et al. 2009). *Xenopus* eggs were collected and dejellied with 2.2% (w/v) cysteine hydrochloride (pH 7.7 with NaOH), washed three times with 0.5× modified MMR (50 mM NaCl, 1 mM KCl, 0.5 mM $MgSO_4$, 1 mM $CaCl_2$, 0.05 mM ethylenediaminetetraacetic acid [EDTA], 25 mM Hepes-NaOH, pH 7.8), and three times with 1× egg lysis buffer (ELB: 10 mM Hepes-KOH, 2.5 mM $MgCl_2$, 50 mM KCl, pH 7.7) containing 250 mM sucrose, 1 mM dithiothreitol (DTT), and 50 µg/mL cycloheximide. Eggs were then packed in 50-mL polycarbonate tubes (Thermo Fisher Scientific, Waltham, MA, USA, Cat#3117-0500) and crushed by centrifugation at 10,000 rpm for 20 min in Tomy NS-1 (TOMY Seiko, Tokyo, Japan) after removing excess buffers and adding 2.5 µg/mL cytochalasin B, 5 µg/mL aprotinin, and 5 µg/mL leupeptin. Crude cytoplasmic extracts were recovered, supplemented with 5 µg/mL cytochalasin B, 10 µg/mL aprotinin, 10 µg/mL leupeptin, 50 µg/mL cycloheximide, 1 mM DTT, and 3.3 µg/mL nocodazole, and re-centrifuged at 30,000 rpm for 30 min in Beckman SW50.1 (Beckman Coulter, Brea, CA, USA). Cleared extracts were recovered, supplemented with 2 mM adenosine triphosphate (ATP), 20 mM phosphocreatine, 5 µg/mL creatine phosphokinase, and 4,000 /µL demembraned sperm nuclei, incubated at 22°C for 100–120 min, and centrifuged at 9,000 rpm for

2 min in NS-1. The layer of nuclei floating on the top of the extract was collected and centrifuged at 46,000 rpm for 30 min in SW50.1. Nucleoplasmic extracts separated from lipids and chromatin were then collected, frozen in liquid nitrogen as 10–20 µL aliquots, and stored at -80°C.

Preparation of MMR substrates

Oligonucleotides used in this study are listed in Supplemental Table S5. For the primer-extension assay, oligonucleotides 406 and 407 were used for the no mismatch and A:C-mismatch-carrying primers, respectively.

In vitro synthesis of mismatch-carrying plasmids was performed essentially as described previously (Higashi et al. 2012; Kawasoe et al. 2016). Briefly, an oligonucleotide DNA was annealed on single-stranded phagemid DNA, and the complementary DNA strand was synthesized by T7 DNA polymerase (New England Biolabs, Ipswich, MA, USA). After ligation of remaining nicks by T4 DNA ligase (Nippongene, Tokyo, Japan), covalently closed circular DNA molecules were purified over cesium chloride/ethidium bromide density gradient centrifugation. To introduce mismatches, the following oligonucleotides were used: 721 for control homoduplex, 722 for an A:C mismatch, 411 for a ±1 insertion/deletion loop (IDL), 412 for a ±5 IDL, 414 for a G:G mismatch, and 415 for a T:C mismatch. To introduce a site-specific biotin modification, 362 was also used. To introduce two additional mismatches, following oligonucleotides pairs were also used: 723 and

725 for control homoduplex, and 724 and 726 for an A:C and a T:C mismatch. A site-specific gap was introduced using Nt.BbvCI (New England Biolabs), as described previously (Kawasoe et al. 2016).

Yeast strains

All *S. cerevisiae* strains used in this study were derived from BY4741 and are listed in Supplemental Table S4. Transformation was performed using the standard lithium acetate method (Gietz and Woods 2002). The *hom3-10* and the *lys2::insE-A14* mutations were introduced as described below: Two fragments of the *hom3-10* gene were individually amplified by PCR from BY4741 genomic DNA with primers 1304 and 1315, and 1305 and 1318, respectively. The fragments were then fused by overlap-extension PCR with primers 1315 and 1318 and directly used for transformation of TTY15, in which the *HOM3* gene was disrupted by the *URA3* gene. Clones carrying the *hom3-10* mutation were selected on complete media (synthetic complete: SC) containing 5-fluoroorotic acid. The *lys2::insE-A14* allele was introduced into the resulting *hom3-10* strain (TTY20) by integration of the YIpURA3-*lys2::insE-A14* plasmid linearized by XhoI (New England Biolabs) and excision of *URA3* and the wild-type *LYS2* gene. Further genetic manipulation was carried out using following plasmids or PCR fragments with following primers: *msh2*, 1347, 1348, 1349, and 1350; *msh6*, 1359, 1360, 1361, and 1362; *msh3*, 1365, 1366, 1367, and 1368; *fun30*, 1353, 1354, 1355, and 1356; *fun30-K603A*,

YIpURA3-*fun30*-K603A (linearized with PstI [New England Biolabs]); *cac1*, 1537, 1538, 1539, and 1540; *exo1*, 1481, 1482, 1483, and 1484; *rad52*, 1457, 1458, 1459, and 1460; *spt16-d922*, YIpURA3-*spt16-d922* (linearized with SalI [New England Biolabs]). After each transformation step, gene integration was verified by colony-directed PCR. For integration of a point mutation, the sequence of the entire gene was confirmed after PCR amplification.

Cloning and plasmids

Construction of pMM1 was described previously (Kawasoe et al. 2016). Construction of pMM3 was performed as follows: A linker DNA fragment was amplified by PCR with primers 1079 and 1158 using fission yeast genomic DNA as a template. The DNA fragment was digested with PstI and BspQI (New England Biolabs) and inserted between the same sites in pMM1, resulting in pMM3. Cloning of *Xenopus laevis smarcad1* gene was performed as follows: A BLAST search using the *Xenopus tropicalis* Smarcad1 sequence identified two *Xenopus laevis* EST clones, TC422950 and TC460920. Based on these EST sequences, we designed two primers, 900 and 887, and amplified the *smarcad1* gene by PCR from *Xenopus* egg cDNA. The *smarcad1* gene fragment was digested with NdeI and BamHI-HF (New England Biolabs) and cloned into pDE1a, a derivative of the pDONR201 vector (Life Technologies, Carlsbad, CA, USA) carrying NdeI and BamHI sites between attL1 and L2 sites. Sequencing of cloned genes revealed

that two distinct isoforms, which we named *smarcad1a* and *smarcad1b*, were present (Plasmids: pDE1a-SMARCAD1A and pDE1a-SMARCAD1B). Smarcad1a and Smarcad1b were 90% identical and 95% similar with respect to their amino acid sequences. The *smarcad1a* gene was used for all subsequent construction and experiments, and therefore the gene product was called simply Smarcad1, unless otherwise indicated. To introduce the lysine 503 to alanine substitution in the Walker A motif, the gene fragment was amplified by PCR using primer pairs, 887 and 955, and 900 and 956, using pDE1a-SMARCAD1A as a template, and the two PCR fragments were fused by overlap-extension PCR with primers 887 and 900. The resulting *smarcad1a*^{K503A} fragment was digested with NdeI and EcoRI (New England Biolabs), and cloned into the same sites in pDE1a-SMARCAD1A, resulting in pDE1a-SMARCAD1A-K503A. To add two tandem FLAG tags to the N-terminus of Smarcad1, a synthetic linker prepared by annealing of 5′-phosphorylated oligonucleotides 60 and 61 was inserted in the NdeI sites in pDE1a-SMARCAD1A and pDE1a-SMARCAD1A-K503A, resulting in pDE1a-FLAG-SMARCAD1A and pDE1a-FLAG-SMARCAD1A-K503A, respectively. Baculoviruses for expression of FLAG-Smarcad1 and FLAG-Smarcad1-K503A were prepared by transferring the FLAG-*smarcad1a* and FLAG-*smarcad1a*^{K503A} genes into BaculoDirect C-term Linear DNA (Life Technologies) using the Gateway LR reaction.

cDNAs of *Xenopus laevis spt16* and *ssrp1* genes were kind gifts from Haruhiko Takisawa, Yumiko Kubota, and Masato Kanemaki. The *spt16* gene was amplified by two-step PCR using primers 798 and 799, and then primers 344 and 345, and cloned into the pDONR201 vector using the Gateway BP reaction, resulting in pDONR-SPT16. The *ssrp1* gene was amplified by PCR using primers 770 and 771, digested with NcoI (New England Biolabs) and Sse8387I (Takara, Kusatsu, Japan), and cloned into the same sites in a modified pDE1a vector, resulting in pDONR-SSRP1. For protein expression in *Escherichia coli*, the gene fragments on the Gateway entry vectors were transferred into pET-HSD, a derivative of the pETDuet-1 vector (Merck Millipore, Billerica, MA, USA, Cat#71146-3CN) carrying a Gateway recombination cassette and a His-tag for N-terminal fusion, by the Gateway LR reaction, resulting in pET-HSD-SPT16 and pET-HSD-SSRP1, respectively. The N-terminally His_6-FLAG-tagged *spt16* gene was amplified by two-step PCR using primers 799 and 827, and then primers 799 and 81, digested with NcoI, and cloned into pDONR-SPT16, resulting in pDONR-His_6-FLAG-SPT16. Baculoviruses for expression of His_6-FLAG-Spt16 and Ssrp1 were constructed by transferring the His_6-FLAG-*spt16* and *ssrp1* genes into BaculoDirect C-term Linear DNA by the Gateway LR reaction.

Cloning of the *Xenopus laevis msh3* gene was performed as follows: A BLAST search using the *Xenopus tropicalis* Msh3 sequence identified a partial *Xenopus laevis* EST clone, CA988114. The missing 5′ and 3′ portions of the *msh3*

cDNA were cloned by 5′ and 3′ RACE using the SMARTer RACE cDNA Amplification kit (Clontech, CA, USA) with primers 784 and 780, respectively. The full-length *msh3* ORF was then PCR-amplified from *Xenopus laevis* egg cDNA by using primers 957 and 958, and then 344 and 355, and cloned into pDONR201 by the Gateway BP reaction, resulting in pDONR-MSH3. For protein expression in *E. coli*, the *msh3* gene was transferred into pET-HSD by the Gateway LR reaction, resulting in pET-HSD-MSH3.

The budding yeast *fun30-K603A* mutant gene in which lysine 603 in the Walker A motif was replaced with alanine was prepared by overlap-extension PCR with primers 1564, 1565, 1566, and 1567 using BY4741 genomic DNA as templates. The resulting fragment was digested with EcoRI and BamHI, and cloned into YIplac211, resulting in YIpURA3-fun30-K603A.

The *lys2::insE-A14* gene was constructed as follows: Two partially overlapping fragments of the *lys2::insE-A14* gene were separately prepared by two-step PCR with following primer pairs: the 5′ half of the fragment, 1296 and 1298, and 1296 and 1426; the 3′ half of the fragment, 1297 and 1301, and 1297 and 1300. Two fragments were then simultaneously inserted into pBluescript II KS(-) linearised by PCR with primers 1294 and 1295 by the Gibson assembly reaction (New England Biolabs), resulting in pBS-lys2::insE-A14. The PvuII-PstI fragment of pBS-lys2::insE-A14 was subcloned between the PstI and SmaI sites in YIplac211, resulting in YIpURA3-lys2::insE-A14.

The *spt16-d922* mutant gene was prepared by two-step overlap-extension PCR with primers 1571, 1572, 1573, and 1574 using BY4741 genomic DNA as templates. The fragment was digested with BamHI and HindIII (New England Biolabs), and cloned into YIplac211, resulting in YIpURA3-spt16-d922.

Construction of pDONR-xMLH1 was described previously (Kawasoe et al. 2016). For protein expression in *E. coli*, the *mlh1* gene was transferred into pDEST17 (Life Technologies) by the Gateway LR reaction, resulting in pDEST17-MLH1.

Protein expression and purification

Purification of *Xenopus laevis* MutSα was carried out as described previously (Kawasoe et al. 2016).

Purification of *Xenopus laevis* Smarcad1 was performed as follows: Recombinant protein was expressed by infecting Sf9 insect cells with FLAG-Smarcad1 baculoviruses at 28°C in Sf-900II SFM (Life technologies) supplemented with 2% (v/v) fetal bovine serum. Cells were harvested, washed with phosphate buffered saline (PBS), and frozen in liquid nitrogen. Cells were suspended in buffer S (25 mM Tris-HCl, 10% glycerol, 300 mM NaCl, 5 mM 2-mercaptoethanol, 1 mM EDTA, pH 7.4) containing 1x cOmplete EDTA-free (Roche Life Science, Penzberg, Germany), and the lysates were centrifuged at 81,800 *xg* (30,000 rpm) for 30 min in Beckman 50.2Ti (Beckman Coulter). Cleared lysates were passed through

FLAG-M2 agarose (Sigma Aldrich, St. Louis, MO, USA). The FLAG-Smarcad1 protein was eluted from the FLAG-M2 resin with 50 µg/mL FLAG-peptide (Sigma Aldrich) in buffer S containing 0.1x cOmplete EDTA-free. Peak fractions were pooled and three-fold diluted with buffer A (20 mM Tris-HCl, 5% glycerol, 5 mM 2-mercaptoethanol, 1 mM EDTA, pH 7.4) containing 0.1x cOmplete EDTA-free, loaded on a MonoQ 5/50 GL column (GE Healthcare, Little Chalfont, UK), and the column was developed with a 0–1 M NaCl linear gradient in buffer A containing 0.1x cOmplete EDTA-free. Peak fractions were pooled and loaded on a Hi Load 16/60 Superdex 200 prep grade column (GE Healthcare), and the column was developed with buffer A containing 0.14 M NaCl. Fractions corresponding to the molecular mass of $2.5–5.0 \times 10^5$ (FLAG-Smarcad1: $M_r = 1.19 \times 10^5$) were pooled, concentrated using Amicon Ultra (Merck Millipore), and frozen in liquid nitrogen as small aliquots.

Purification of the N-terminally His_6-tagged, full-length *X. laevis* Msh3 protein was performed as follows: Protein expression was induced in *E. coli* BL21(DE3) transformed with pET-HSD-MSH3 by addition of 0.4 mM isopropyl-β-D-thiogalactopyranoside (IPTG) for 1 h at 37°C. Cells were harvested, lysed with 1 mg/mL lysozyme and sonicated in buffer SO (50 mM Na-phosphate, 500 mM NaCl, 1% Triton X-100, pH 8.0) containing 1 mM phenylmethylsulfonyl fluoride (PMSF) and 2 mM benzamidine and centrifuged at 10,000 rpm for 10 min in TA-24BH (TOMY Seiko). The inclusion bodies containing the Msh3 protein were

resuspended in buffer SO, centrifuged again at 10,000 rpm for 10 min in TA-24BH, and these procedures were repeated three times. The Msh3 protein was dissolved in Laemmli's SDS sample buffer (62.5 mM Tris-HCl, 10% glycerol, 3% SDS, 0.005% bromophenol blue, 5% 2-mercaptoethanol, pH 6.8) and purified by SDS-PAGE followed by electroelution.

Purification of the *X. laevis* FACT heterodimer was performed as follows: Recombinant proteins were expressed by co-infecting Sf9 insect cells with His_6-FLAG-Spt16 and Ssrp1 baculoviruses at 28°C in Sf-900II SFM supplemented with 2% (v/v) fetal bovine serum. Cells were harvested, washed with PBS and frozen in liquid nitrogen. Cells were suspended in buffer S containing 2 mM PMSF and 1 mM benzamidine and centrifuged at 81,800 *xg* (30,000 rpm) for 30 min in Beckman 50.2Ti. Cleared lysates were passed through a DEAE Sepharose Fast Flow column (GE Healthcare) and then a FLAG-M2 agarose column. The FACT heterodimer was eluted from the FLAG-M2 resin with 50 µg/mL FLAG-peptide in buffer S containing 0.2 mM PMSF and 0.1 mM benzamidine. Peak fractions were pooled and diluted three-fold with buffer A containing 0.2 mM PMSF and 0.1 mM benzamidine, loaded on a HiTrap Q-HP 1-mL column (GE Healthcare), and bound proteins were eluted with a 0–1 M NaCl linear gradient in buffer A. Peak fractions were pooled, dialyzed against buffer D (20 mM Tris-HCl, 100 mM KCl, 5% glycerol, pH 7.4), concentrated by Amicon Ultra, and frozen in liquid nitrogen as small aliquots.

Purification of the N-terminally His_6-tagged, full-length *X. laevis* Spt16 protein was performed as follows: Spt6-containing inclusion bodies were purified by the method essentially the same as that for Msh3, except that protein expression was induced for 2 h. The inclusion bodies were resuspended in 0.5× buffer SO containing 0.5 mM PMSF, 1 mM benzamidine, 7 M urea, 2 M thiourea, 100 mM DTT. 4× Laemmli's SDS sample buffer was also added to final 1× concentration. The sample was incubated for 20 min at 37°C and centrifuged at 15,000 rpm for 20 min in TA-24BH to remove insoluble debris. The Spt16 protein was then purified by SDS-PAGE followed by electroelution.

Purification of the N-terminally His_6-tagged, full-length *X. laevis* Ssrp1 protein was performed as follows: The method for protein expression and preparation of bacterial lysate were essentially the same as that for Msh3, except that protein expression was induced at 20°C for 20 h. The lysate was centrifuged at 81,800 *×g* (30,000 rpm) for 30 min in Beckman 50.2Ti. The His-Ssrp1 protein in the cleared lysate was bound to the TALON metal affinity resin (Clontech) for 1 h at 4°C and eluted with 100 mM imidazole in buffer W (20 mM Na-phosphate, 500 mM NaCl, 0.1% Triton X-100, pH 8.0) containing 0.1 mM PMSF and 0.2 mM benzamidine. The eluate was diluted four-fold with buffer B (50 mM Na-phosphate, 5% glycerol, pH 6.8), loaded on a HiTrap Q-HP 1-mL column, and the column was developed with a 0–1 M NaCl linear gradient in buffer B. Peak fractions were pooled, diluted four-fold with buffer B, loaded on a HiTrap SP-HP 1-mL column (GE

Healthcare), and the column was developed with a 0–1 M NaCl linear gradient in buffer B.

The *E. coli* BL21 codon plus (DE3) cells carrying pET28c-xHIRA was a kind gift from Masato Kanemaki. The method for expression and purification of the *X. laevis* HIRA protein was essentially the same as that for Msh3, except that protein expression was induced for 7 h at 37°C.

Purification of the N-terminally His_6-tagged, full-length *X. laevis* Mlh1 protein was performed as follows: The method for protein expression and preparation of bacterial lysate were essentially the same as that for Msh3, except that protein expression was induced for 5 h. Inclusion bodies containing the Mlh1 protein were resuspended in wash buffer (50 mM Na-phosphate, 1 M NaCl, 0.1% Triton X-100, pH 8.0) and centrifuged at 13,000 rpm for 20 min in TA-24BH. The pellet was resuspended in wash buffer containing 1 M urea, centrifuged again at 13,000 rpm for 20 min in TA-24BH, and these procedures were repeated three times. The Mlh1 protein was dissolved in Laemmli's SDS sample buffer containing 4 M urea and purified by SDS-PAGE followed by electroelution.

Immunological methods

Production and usage of Msh2R1, Msh6, Mlh1 (Kawasoe et al. 2016), and Cdc7 antibodies (Takahashi and Walter 2005) were described previously. The rabbit Msh2pep antiserum was raised against peptide NH_2-CLAKNNRFVSEVISRTKTGL-

COOH, corresponding to residues 914–932 of Msh2. The rabbit Msh2R2 antiserum was raised against N-terminally His_6-tagged and C-terminally Strep-II-tagged full-length Msh2 expressed in *E. coli*. The rabbit Msh3 antiserum was raised against N-terminally His_6-tagged, full-length Msh3 expressed in *E. coli*. The rabbit HIRA antiserum was raised against N-terminally His_6-tagged, full-length HIRA expressed in *E. coli*. The rabbit Spt16 antiserum was raised against N-terminally His_6-tagged, full-length Spt16 expressed in *E. coli*. The rabbit Ssrp1 antiserum was raised against N-terminally His_6-tagged, full-length Ssrp1 expressed in *E. coli*. The rabbit xH2B antiserum was raised against peptide NH_2-CAKHAVSEGTKAVTKYTSAK-COOH, corresponding to residues 108–126 of H2B. The rabbit xH3 antiserum was raised against peptide NH_2-ARTKQTARKSTGGKAC-COOH and NH_2-CPKDIQLARRIRGERA-COOH, corresponding to residues 1–15 and 121–135 of H3, respectively. The rabbit Smarcad1 antiserum was raised against peptide NH_2-CDEGTIPLDMATLLKTSLGL-COOH, corresponding to residues 983–1001 of Smarcad1a. This peptide is 100% conserved between Smarcad1a and Smarcad1b, and therefore the resulting antibodies should recognize both isoforms. The rabbit xCAF-1 antiserum was raised against peptide NH_2-CSSADKPSGSDQTNK-COOH and NH_2-CFDEIKKRKPRKMG-COOH, corresponding to residues 555–569 of xCAF-1 p60 and 450–452 of xCAF-1 p150, respectively. All antibodies except for Mlh1, Spt16, Ssrp1, and CAF-1 were affinity-purified using corresponding antigens. The rabbit Orc2 antiserum was a kind gift from Johannes Walter (Vashee et al. 2003).

The mouse histone H4 monoclonal antibody was a kind gift from Hiroshi Kimura (Hayashi-Takanaka et al. 2015). The CAF-1 p150 and p60 antibodies used for immunoblots in Fig. S5A were kind gifts from Ruibin Zhu, Mari Iwabuchi, and Keita Ohsumi (Zhu et al. 2017). For immunoblotting, Msh2, Msh6, Mlh1, Smarcad1, Spt16, Ssrp1, p150, p60, xH3, H4 and Orc2 antisera were used at a dilution of 1:5,000. For immunoblotting of HIRA, xH2B, and Msh3, affinity-purified antibodies were used at 0.5–1 µg/ml. HRP-conjugated Goat Rabbit IgG (H+L) antibodies (Jackson ImmunoResearch, West Grove, PA, USA, Cat#111-035-003), or Goat Mousse IgG (H+L) antibodies (#115-035-146) were used at a dilution of 1:10,000 as the secondary antibody. The secondary antibodies were detected by enhanced chemiluminescence using the SuperSignal West Pico or West Femto Chemiluminescent Substrate (Thermo Fisher Scientific), and the signals were collected either by ImageQuant LAS500 (GE Healthcare) or ChemiDoc Touch (BioRad Laboratories).

For immunoprecipitation, 3 vol of an antiserum was bound to 1 vol of recombinant protein A-Sepharose (PAS, GE Healthcare). NPE was diluted 5-fold with ELB, and centrifuged at 15,000 rpm for 10 min in a benchtop centrifuge to remove insoluble debris. For each immunoprecipitation reaction, 12 µL of diluted NPE was mixed with 4 µL of IgG-coupled PAS, incubated at 4°C for 2 h with gentle rotation, and the supernatant and the beads were separated by centrifugation at 5,000 rpm for 30 sec in a benchtop centrifuge. The beads were washed three times

with ELB containing 0.1% Triton X-100, and the bound proteins were eluted with 20 µL of Leammli's SDS sample buffer.

Immunodepletion was performed as follows: For Smarcad1, 5 vol of the Smarcad1 serum was bound to 1 vol of PAS. For Spt16, Mlh1, Msh3, or CAF-1, 3 vol of the serum was bound to 1 vol of PAS. For Spt16/Smarcad1-double depletion, 13 µg of xSmarcad1 IgG and 3 µL of the Spt16 serum were bound to 1 µL of PAS. For Msh2/Smarcad1-double depletion, 13 µg of xSmarcad1 IgG was bound to 1 µL of PAS. For HIRA, 20 µg of xHIRA IgG was bound to 1 µL of PAS. For Msh6, 0.5 µg of Msh6 IgG and 3 µL of the Msh6 serum were bound to 1 µL of PAS. For MutSα/MutSβ depletion from NPE, 2 µg of Msh2R1 IgG, 0.5 µg of Msh6 IgG, and 3 µL of the Msh6 serum were bound to 1 µL of PAS. To deplete NPE, 0.2 vol of IgG-coupled PAS was mixed with 1 vol of NPE, incubated at 4°C for 1 h with gentle rotation, and the procedure was repeated twice except for Mlh1-depletion, in which the procedure was repeated once. For depletion of Spt16 or double-depletion of Spt16/Smarcad1, 0.3 instead of 0.2 vol of IgG-coupled PAS was used. For depletion of HIRA, 0.1 vol of HIRA-IgG coupled PAS was used. For double-depletion of Msh2/Smarcad1, 0.15 vol of Msh2/Msh6-IgG coupled PAS and 0.15 vol of Smarcad1-IgG coupled PAS were used. In most cases, 20–60 µL of NPE was depleted for each experiment.

Determination of the mutation spectra

Mutation sites were determined by Sanger sequencing of target regions amplified by colony-directed PCR. The *hom3* locus was amplified with primers 1290 and 1293, and sequenced with 1290, and the *lys2* locus was amplified with primers 1311 and 1314, and sequenced with 1428 and 1429. To ensure that each of the reversion mutations had been independently arisen, only one reversion mutant was isolated from an independent culture for sequencing.

Statistical Testing

At least two biological replicates, in each of which 7–11 technical replicates were included, were performed for each strain to estimate reliably the reversion rates (Rosche and Foster 2000). The exact number of total replicates (*n*), including both biological and technical replicates, is as follows: wild-type, 30; *fun30Δ*, 30; *fun30-K603A*, 25; *msh2Δ*, 22; *msh2Δ fun30Δ*, 22; *msh6Δ*, 22; *msh6Δ fun30Δ*, 22; *msh6Δ fun30-K603A*, 30; *msh3Δ*, 22; *msh3Δ fun30Δ*, 22 for *hom3* and 21 for *lys2*; *exo1Δ*, 37; *exo1Δ fun30Δ*, 37; *exo1Δ msh6Δ*, 22; *exo1Δ msh6Δ fun30Δ*, 22; *rad52Δ*, 30; *rad52Δ fun30Δ*, 29; *rad52Δ msh6Δ*, 21 for *hom3* and 22 for *lys2*; *rad52Δ msh6Δ fun30Δ*, 22; *cac1Δ*, 22; *fun30Δ cac1Δ*, 35 for *hom3* and 20 for *lys2*; *msh6Δ cac1Δ*, 22; *msh6Δ fun30Δ cac1Δ*, 22; *msh3Δ cac1Δ*, 22; *msh3Δ fun30Δ cac1Δ*, 22; *msh2Δ cac1Δ*, 34 for *hom3* and 33 for *lys2*; *spt16-d922*, 30; *spt16-d922 fun30Δ*, 30; *spt16-d922 msh6Δ*, 38; *spt16-d922 msh6Δ fun30Δ*, 24.

To obtain the *p*-values, the number of revertants obtained by the same

procedure was normalized by using viable cell counts, and compared by Mann-Whitney's U-test. Calculation was performed using Graphpad Prism 6 (Graphpad Software, La Jolla, CA, USA).

Repeatability

For supercoiling assays, mismatch-DNA binding assays, immunoprecipitations, and micrococcal nuclease digestion experiments, representative results, out of at least three independent experiments using at least two different preparations of NPE, are shown. Immunoblots for evaluation of depletion efficiencies were carried out once for each single depletion experiment. Spectral counting by mass spectrometry was carried out three times using three independent samples. Because there was no reliable method to merge spectral counts obtained from different experiments, two representative data were presented.

Code availability

Calculation of the MSS maximum likelihood function was performed by using a custom software coded by using Swift2.0 on Xcode (Apple Computer, Cupertino, CA, USA). The source code and the compiled software will be provided upon request.

Data availability

The GenBank accession numbers for sequences of *Xenopus smarcad1a*, *smarcad1b*, and *msh3* mRNA reported in this paper are LC183875, LC183876, and LC183877, respectively. All data supporting the findings of this study are available from the corresponding author on request.

Supplemental References

Gietz RD, Woods RA. 2002. Transformation of yeast by lithium acetate/single-stranded carrier DNA/polyethylene glycol method. *Methods Enzymol* **350**: 87-96.

Hayashi-Takanaka Y, Maehara K, Harada A, Umehara T, Yokoyama S, Obuse C, Ohkawa Y, Nozaki N, Kimura H. 2015. Distribution of histone H4 modifications as revealed by a panel of specific monoclonal antibodies. *Chromosome Res* **23**: 753-766.

Higashi TL, Ikeda M, Tanaka H, Nakagawa T, Bando M, Shirahige K, Kubota Y, Takisawa H, Masukata H, Takahashi TS. 2012. The prereplication complex recruits XEco2 to chromatin to promote cohesin acetylation in Xenopus egg extracts. *Curr Biol* **22**: 977-988.

Kawasoe Y, Tsurimoto T, Nakagawa T, Masukata H, Takahashi TS. 2016. MutSalpha maintains the mismatch repair capability by inhibiting PCNA unloading. *Elife* **5**.

Lebofsky R, Takahashi T, Walter JC. 2009. DNA replication in nucleus-free Xenopus egg extracts. *Methods Mol Biol* **521**: 229-252.

Rosche WA, Foster PL. 2000. Determining mutation rates in bacterial populations. *Methods* **20**: 4-17.

Takahashi TS, Walter JC. 2005. Cdc7-Drf1 is a developmentally regulated protein

kinase required for the initiation of vertebrate DNA replication. *Genes Dev* **19**: 2295-2300.

Vashee S, Cvetic C, Lu W, Simancek P, Kelly TJ, Walter JC. 2003. Sequence-independent DNA binding and replication initiation by the human origin recognition complex. *Genes Dev* **17**: 1894-1908.

Zhu R, Iwabuchi M, Ohsumi K. 2017. The WD40 Domain of HIRA Is Essential for RI-nucleosome Assembly in Xenopus Egg Extracts. *Cell Struct Funct* **42**: 37-48.

Supplemental figure legends

Supplemental Figure S1. Mismatch-dependent exclusion of nucleosomes in NPE

(A) The effect of various types of mismatches on supercoiling in NPE. pMM1 carrying no mismatch, an A:C, T:C, G:G mismatch, or an insertion/deletion loop (IDL) of 1 extrahelical nucleotide (1IDL) was incubated in NPE and sampled at the indicated times. The A:C, T:C, G:G mismatches and 1IDL strongly inhibited supercoiling.

(B–D) qPCR-quantification of undigested DNA after MNase treatment. The DNA amount relative to the input (B), normalized to pControl (C), and normalized to pMM1^{homo} (D) is shown. Mean ± 1SD (n = 3). See Fig. 1D for detail.

(E) The MNase digestion assay was performed with a larger plasmid substrate (pMM3^{AC}). Positions of primers for qPCR are presented on a map of pMM3^{AC}. The DNA amount normalized to the homoduplex DNA (pMM3^{homo}) is presented as a graph. Mean ± 1SD (n = 3). The area with strong nucleosome exclusion was not significantly extended on a larger plasmid compared to the 3-kb substrate.

Supplemental Figure S2. The effect of depletion of MMR proteins on MMR and supercoiling in NPE

(A, B) Immunodepletion of NPE with different Msh2/6 antibodies. Following antibodies were used for depletion: Msh6 (against residues 1324–1340 of Msh6),

Msh2pep (against residues 914–932 of Msh2), and Msh2R1 and Msh2R2 (against full-length Msh2, from different rabbits). Msh2R1 was exclusively used for Msh2 depletion throughout the paper, and therefore it was simply referred to as Msh2 antibodies in other experiments. (A) Depletion efficiencies. 100% corresponds to 0.25-µL NPE. Orc2 served as a loading control. (B) Supercoiling activity of depleted NPE. See Fig. 1B for detail. Depletion with all Msh2/6 antibodies consistently allowed supercoiling of pMM1^{AC}, suggesting that the inhibition of pMM1^{AC} supercoiling depends on the Msh2-Msh6 complex.

(C, D) Gap-directed MMR in Msh6-depleted NPE. (C) Immunodepletion efficiency of Msh6. 0.03 µL each of NPE was loaded. Cdc7 served as a loading control. The depletion efficiency was estimated to be 90%. (D) pMM1^{AC} carrying a 15-nt gap on the A-strand was incubated in NPE described in (C) for 30 min. DNA was purified and digested with XmnI and either XhoI (lanes 1–3, C to T repair) or BamHI (lanes 4–6, A to G repair). *%repair* was calculated based on the percentage of XhoI or BamHI sensitive DNA molecules. The Msh6 depletion strongly inhibited MMR of an A:C mismatch.

(E) Quantification of Msh2 and Msh3 in NPE. NPE was treated with indicated antibodies and the indicated amount of either recombinant MutSα (top) or His-Msh3 (bottom) was supplemented to the extract. 0.2 µl each of NPE was separated by SDS-PAGE and probed with the indicated antibodies. The concentration of Msh2 was estimated to be approximately 1 µM, and that of Msh3 was estimated to be

approximately 6 nM.

(F, G) The effect of MutSβ-depletion on nucleosome exclusion. pMM1 carrying no mismatch, an A:C mismatch, 1IDL, or 5IDL was incubated in mock-treated, MutSα/MutSβ-depleted (with Msh2 and Ms6 antibodies), MutSα(Msh6)-depleted, or MutSβ(Msh3)-depleted NPE and sampled at the indicated times. Depletion efficiencies (F) and the supercoiling assay (G) are presented. Although depletion of MutSα allowed supercoiling of plasmids carrying an A:C mismatch and 1IDL, depletion of MutSβ did not detectably affect the efficiency of supercoiling. Plasmids carrying a 5IDL were supercoiled in all conditions tested, suggesting that 5IDL does not induce significant nucleosome exclusion.

(H) Gap-directed MMR in Mlh1-depleted NPE. The assay presented in (D) was repeated in NPE described in Fig. 2C. The specific A to G repair was completely inhibited by depletion of Mlh1.

(I) Immunodepletion efficiency of Msh2 for Fig. 2F. 100% corresponds to 0.25-µL NPE. The depletion efficiency for Msh2 was estimated as 99%. (*) cross-reacting band.

Supplemental Figure S3. Characterization of Smarcad1 and FACT antisera

The indicated amount of low-speed supernatant (LSS), NPE, or recombinant proteins was separated by SDS-PAGE and transferred onto PVDF membranes. Each membrane strip was probed with either the indicated antiserum or the pre-

immune serum (PI) from the same rabbit. The same exposure sets are presented for each pair of PI and antiserum. Smarcad1 (M_r = 1.14 × 10^5), Spt16 (M_r = 1.18 × 10^5), and Ssrp1 (M_r = 0.79 × 10^5) were detected as nearly a single band in NPE. Each band was specifically immunoprecipitated by the corresponding antibody (see Fig. 4D). (*) cross-reacting band.

Supplemental Figure S4. Smarcad1 assists nucleosome exclusion

(A) Immunodepletion efficiency of Smarcad1 for Fig. 5B. The indicated amount of mock-treated (lanes 1–7) or Smarcad1-depleted NPE (lanes 8–10) supplemented with either buffer (lane 8), 650 nM recombinant $Smarcad1^{WT}$ (lane 9), or $Smarcad1^{K503A}$ (lane 10) was separated by SDS-PAGE and probed with the indicated antibodies. 100% corresponds to 0.25-µL NPE. Orc2 served as a loading control. The depletion efficiency was estimated as 98%. Long exp: long exposure, short exp: short exposure. (*) cross-reacting band.

(B) Recombinant proteins used for the rescue experiments. 1 µg of wild-type (lane 1, WT) or the K503A mutant (lane 2, K503A) of recombinant Smarcad1 purified from baculovirus-infected Sf9 cells were separated by SDS-PAGE and stained with Coomassie brilliant blue R-250.

(C) Immunodepletion efficiency of Smarcad1 for Fig. 5C (Ex #1). The indicated amount of mock-treated (lanes 1, 2, and 5–10) or Smarcad1-depleted NPE (lanes 3 and 4, ΔSm) supplemented with either buffer (lanes 1, 3, 5–10) or 650 nM

recombinant Smarcad1^{WT} (lanes 2 and 4) was separated by SDS-PAGE and probed with the indicated antibodies. 100% corresponds to 0.25-µL NPE. The depletion efficiency was estimated to be higher than 98%. The Smarcad1-depletion efficiency was confirmed to be >98% also for the experiments #2 and #3.

(D) Supercoiling assay in NPE described in (C).

(E) qPCR-quantification of undigested DNA after MNase treatment. The DNA amount normalized to pMM1^{homo} is presented as a graph. See Fig. 5C for detail.

(F, G) Supercoiling assay in Msh2/Smarcad1-doubly-depleted NPE. The depletion efficiencies (F) and the supercoiling assay (G) are presented. The kinetics of plasmid supercoiling were not detectably altered between NPE depleted of Msh2 and that of Msh2 and Smarcad1. These data are in agreement with the hypothesis that Smarcad1 functions downstream of Msh2.

(H, I) Supercoiling assay for mismatch-carrying molecules with BamHI and XhoI digestion. The supercoiling assay presented in Figs. 5D and E was repeated and DNA samples were separated by agarose gel either without restriction enzyme treatment (top) or after digestion with BamHI and XhoI (bottom). Supercoiled plasmids seen in Smarcad1/FACT-depleted NPE retained mismatches, excluding a possibility that depletion of Smarcad1, FACT, or both induces plasmid supercoiling by promoting unregulated correction of the mismatch. The data confirm that depletion of Smarcad1 and FACT enhances supercoiling of the mismatch-carrying molecules.

Supplemental Figure S5. Primer-extension-based chromatin-assembly assay in NPE

(A) Requirement of HIRA and CAF-1 for supercoiling of primer-extension products. NPE was depleted using non-immune (lane 1), HIRA (lane 2), CAF-1 (lane 3), or a mixture of HIRA and CAF-1 antibodies (lane 4). 0.2 µL each of depleted NPE was separated by SDS-PAGE and probed with the indicated antibodies. Orc2 served as a loading control.

(B) Covalently closed $pMM1^{homo}$ (top) or single-stranded pMM1 with a 92-nt primer (bottom) was incubated in the NPE described in (A), sampled at the indicated times, and separated by agarose gel. Although depletion was partial, HIRA-depletion significantly attenuated supercoiling of $pMM1^{homo}$. In contrast, supercoiling of the primer-extension products was inhibited only when both CAF-1 and HIRA were depleted (lanes 11–13).

(C) Immunodepletion efficiency of Msh2 and Mlh1 for Figs. 6B and C. The indicated amount of mock-treated (lanes 1 and 5–9), Mlh1-depleted (lane 2), Msh2-depleted (lane 3), and Msh2/Mlh1-depleted NPE (lane 4) was separated by SDS-PAGE and probed with the indicated antibodies. 100% corresponds to 0.25-µL NPE.

(D) Time-course sampling data of Fig. 6C. Relaxed DNA products were seen in Mlh1-depleted NPE already at 30 min.

(E) Immunodepletion efficiency of Mlh1 and Smarcad1. The indicated amount of

mock-treated (lanes 1 and 5–9), Mlh1- (lane 2), or Mlh1/Smarcad1-depleted NPE (lanes 3 and 4) supplemented with buffer (lanes 1–3) or 650 nM recombinant Smarcad1 (lane 4) was separated by SDS-PAGE and probed with the indicated antibodies. 100% corresponds to 0.25-µL NPE.

(F) Primer-extension assay in Mlh1/Smarcad1-depleted NPE. The assay presented in Fig. 6C was repeated in NPE described in (E).

(G) Immunodepletion efficiency of Mlh1, HIRA, and Smarcad1 for Fig. 6D. The indicated amount of mock-treated (lanes 1 and 5–9), Mlh1/HIRA- (lane 2), or Mlh1/HIRA/Smarcad1-depleted NPE (lanes 3 and 4) supplemented with buffer (lanes 1–3) or 650 nM recombinant Smarcad1 (lane 4) was separated by SDS-PAGE and probed with the indicated antibodies. 100% corresponds to 0.25-µL NPE.

(H) Supercoiling assay in Mlh1/HIRA/Smarcad1-depleted NPE. Closed circular $pMM1^{homo}$ was incubated in NPE described in (G), and sampled at indicated times. Mlh1/HIRA-depletion and Mlh1/HIRA/Smarcad1-depletion significantly attenuated supercoiling of $pMM1^{homo}$.

Supplemental Figure S6. The effect of Smarcad1-depletion on the repair of a mismatch

(A) Immunodepletion efficiencies of Smarcad1 and FACT. NPE was depleted using non-immune (lanes 1 and 5–10; mock), Spt16 (lane 2; ΔFACT), Smarcad1 (lane 3; ΔSmarcad1), or a mixture of Smarcad1 and Spt16 antibodies (lane 4; ΔSmarcad1

ΔFACT). The indicated amount of NPE was separated by SDS-PAGE and probed with the indicated antibodies. 100% corresponds to 0.25-µL NPE.

(B) Gap-directed MMR in the NPE shown in (A). $pMM1^{AC}$ (Top) or $pMM3^{AC}$ (bottom) carrying a 15-nt gap on the A-strand was incubated in the indicated NPE and sampled at the indicated times. DNA was digested with XmnI and BamHI. %repair was calculated based on the percentage of BamHI sensitive DNA molecules. The repair efficiencies were not significantly changed in the absence of Smarcad1, FACT, or both.

(C–E) The effect of Smarcad1-depletion on synthesis-coupled MMR. Single-stranded pMM1 with a 92-nt homoduplex or A:C-mismatch carrying primer was incubated in mock-treated or Smarcad1-depleted NPE and sampled at 120 min. The C to T repair efficiency was estimated by digesting the products with XhoI and XmnI. The depletion efficiencies (C), DNA products (D), and a statistical analysis of replicate experiments (E) are presented. Mean ± 1SD (n = 3). Smarcad1-depletion did not detectably reduce the repair efficiency.

(F, G) Retention of a gap by the p21 peptide in the stepwise-incubation assay. (F) Supercoiling assay in the first NPE. Closed circular $pMM1^{AC}$ (lanes 1 and 4) or $pMM1^{AC}$ carrying 15-nt gap on the A-strand (lanes 2, 3, 5, and 6) was incubated in buffer (lane 3) or Msh2-depleted NPE (lanes 4–6) containing 1 mg/mL p21 PCNA-binding peptide (lanes 4 and 6) for 30 min. Closed circular $pMM1^{AC}$ became mostly supercoiled after incubation in the first NPE (lane 4), suggesting that $pMM1^{AC}$ was

chromatinized by this treatment. After incubation in the first NPE containing the p21 peptide, a majority of pMM1^{AC} carrying 15-nt gap remained in the open circular or relaxed form (lane 6), suggesting that the p21 peptide suppressed gap filling. (G) MMR efficiencies after the incubation in the second NPE. DNA was digested with XmnI and either BamHI (top, A to G repair) or XhoI (bottom, C to T repair). %repair was calculated based on the percentage of XhoI or BamHI sensitive DNA molecules. When gap filling was suppressed by the p21 peptide in the first NPE, the A:C mismatch was efficiently corrected in the second NPE (lanes 14–16).

Supplemental Figure S7. Spectra of reversion mutations of *hom3-10* and *lys2::insE-A14*.

(A) Spectrum of reversion mutations of *hom3-10*. Reversion mutants were sampled and sequenced, and each mutation was plotted on the sequence of the *hom3-10* gene fragment. The run of seven thymines is indicated in red. Underlines indicate simultaneous mutations of multiple bases. Δ indicates a deletion.

(B) Spectrum of reversion mutations of *lys2::insE-A14*.

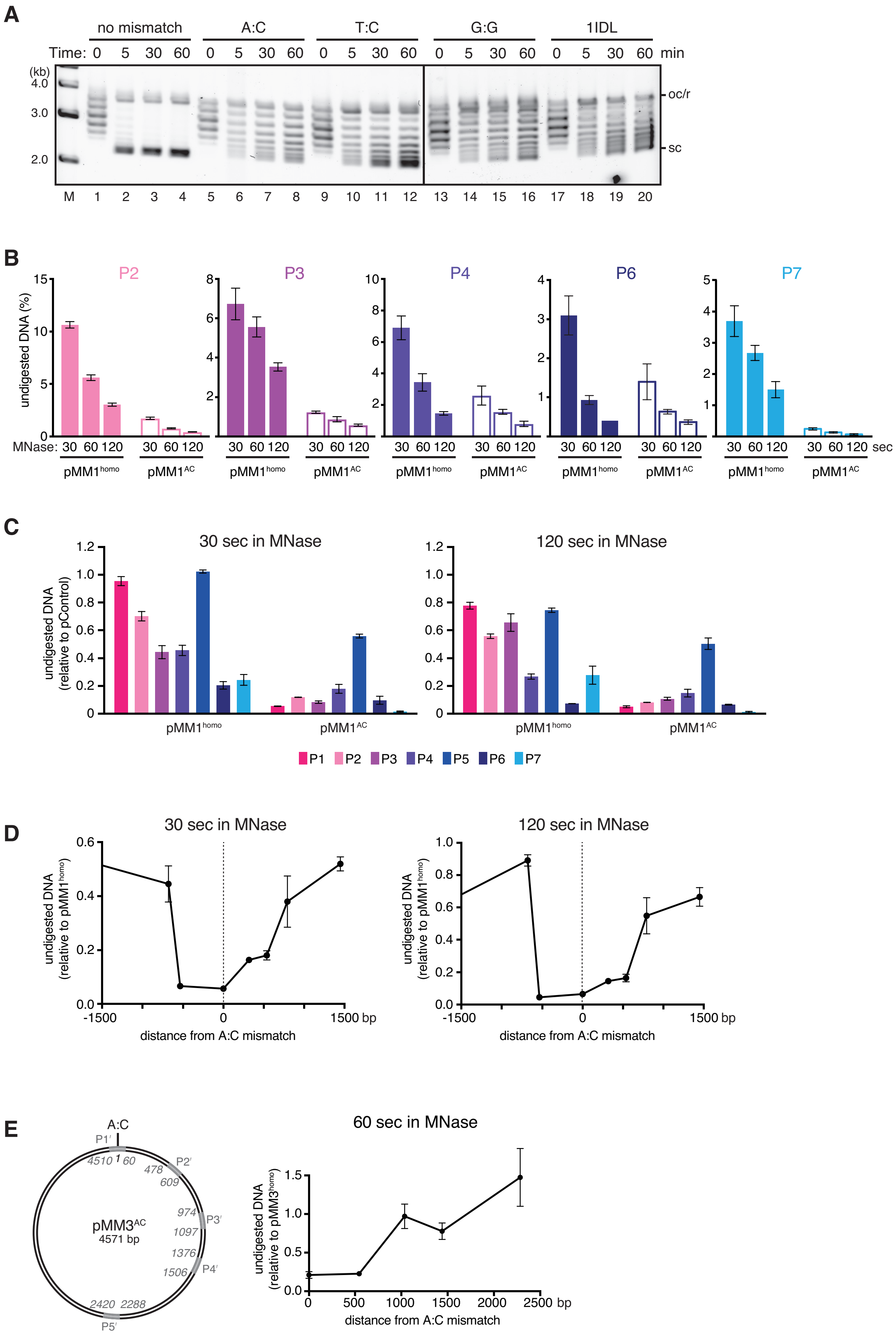
A
no mismatch
A:C
T:C
G:G
1IDL
Time: 0 5 30 60 0 5 30 60 0 5 30 60 0 5 30 60 0 5 30 60 min
(kb)
4.0
3.0
2.0
oc/r
sc
M 1 2 3 4 5 6 7 8 9 10 11 12 13 14 15 16 17 18 19 20
B
P2
P3
P4
P6
P7
undigested DNA (%)
MNase: 30 60 120 30 60 120 sec
pMM1homo
pMM1AC
C
30 sec in MNase
120 sec in MNase
undigested DNA
(relative to pControl)
pMM1homo
pMM1AC
P1 P2 P3 P4 P5 P6 P7
D
30 sec in MNase
120 sec in MNase
undigested DNA
(relative to pMM1homo)
-1500
0
1500 bp
distance from A:C mismatch
E
A:C
P1'
P2'
P3'
P4'
P5'
4510 1 60
478
609
974
1097
1376
1506
2420 2288
pMM3AC
4571 bp
60 sec in MNase
undigested DNA
(relative to pMM3homo)
0 500 1000 1500 2000 2500 bp
distance from A:C mismatch

Terui_Supplemental Figure S2

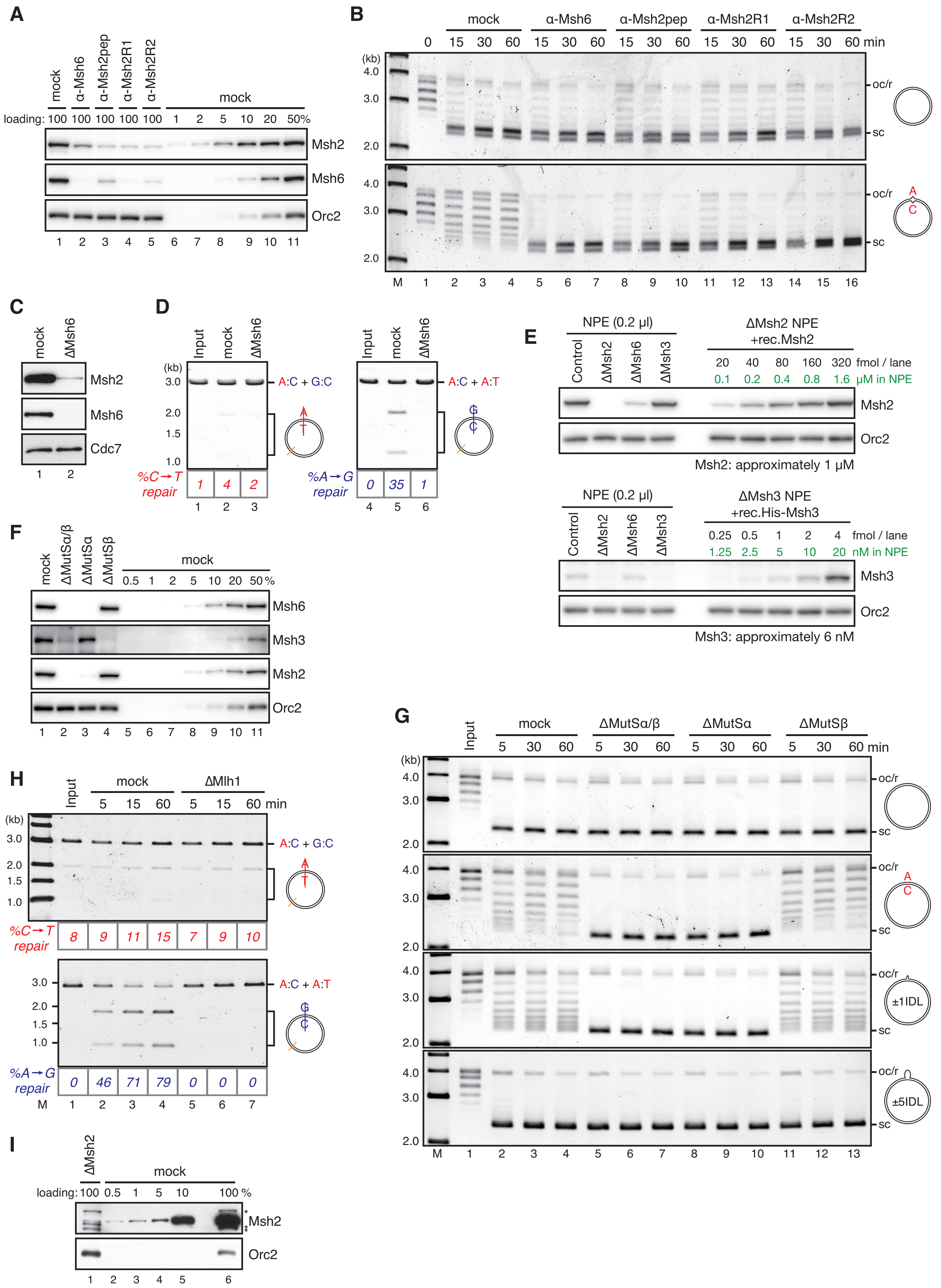

Terui_Supplemental Figure S3

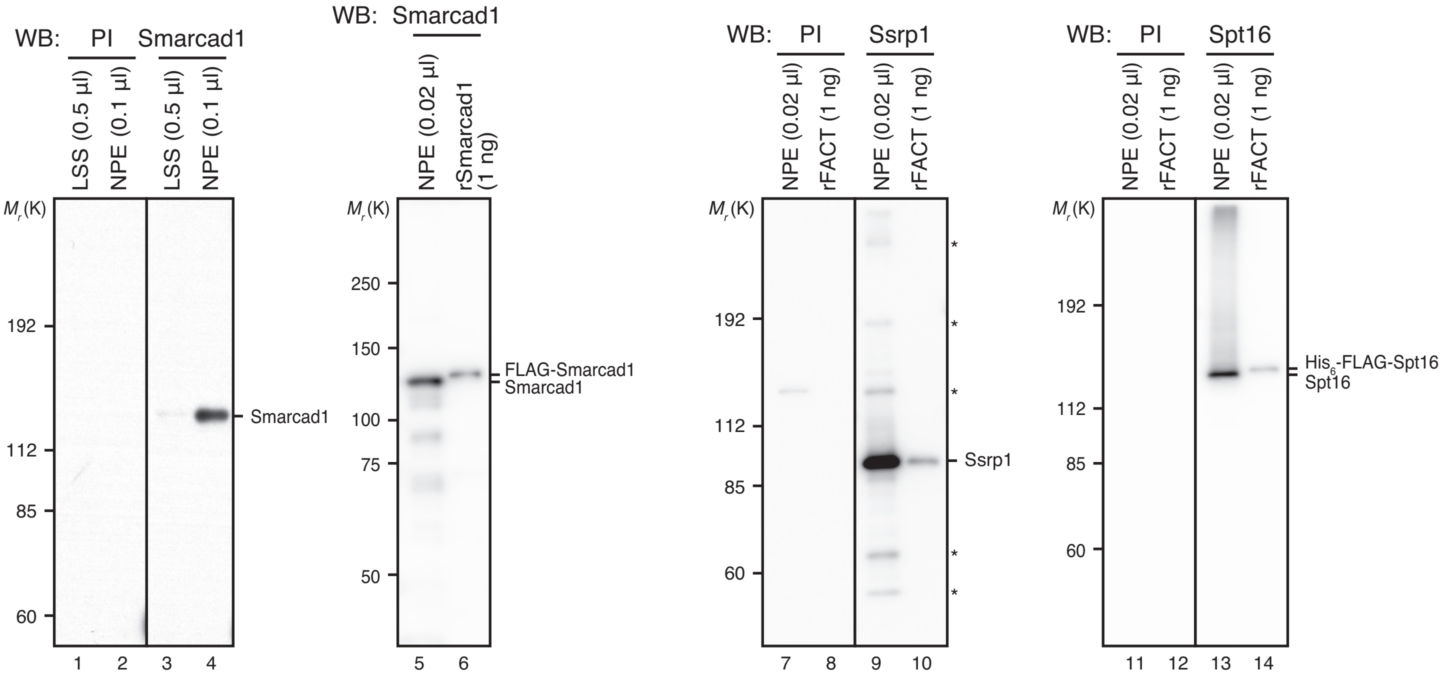

Terui_Supplemental Figure S4

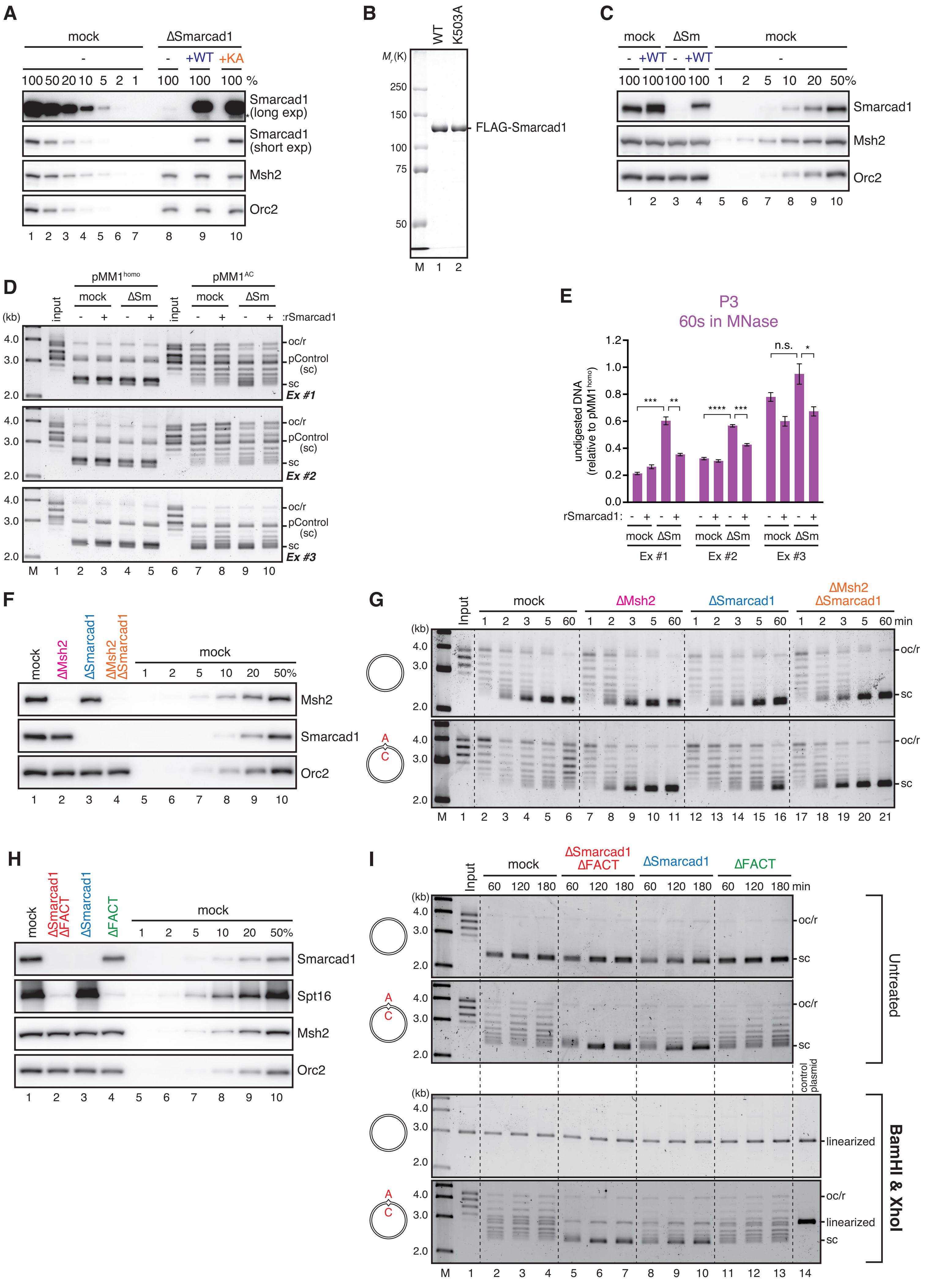

Terui_Supplemental Figure S5

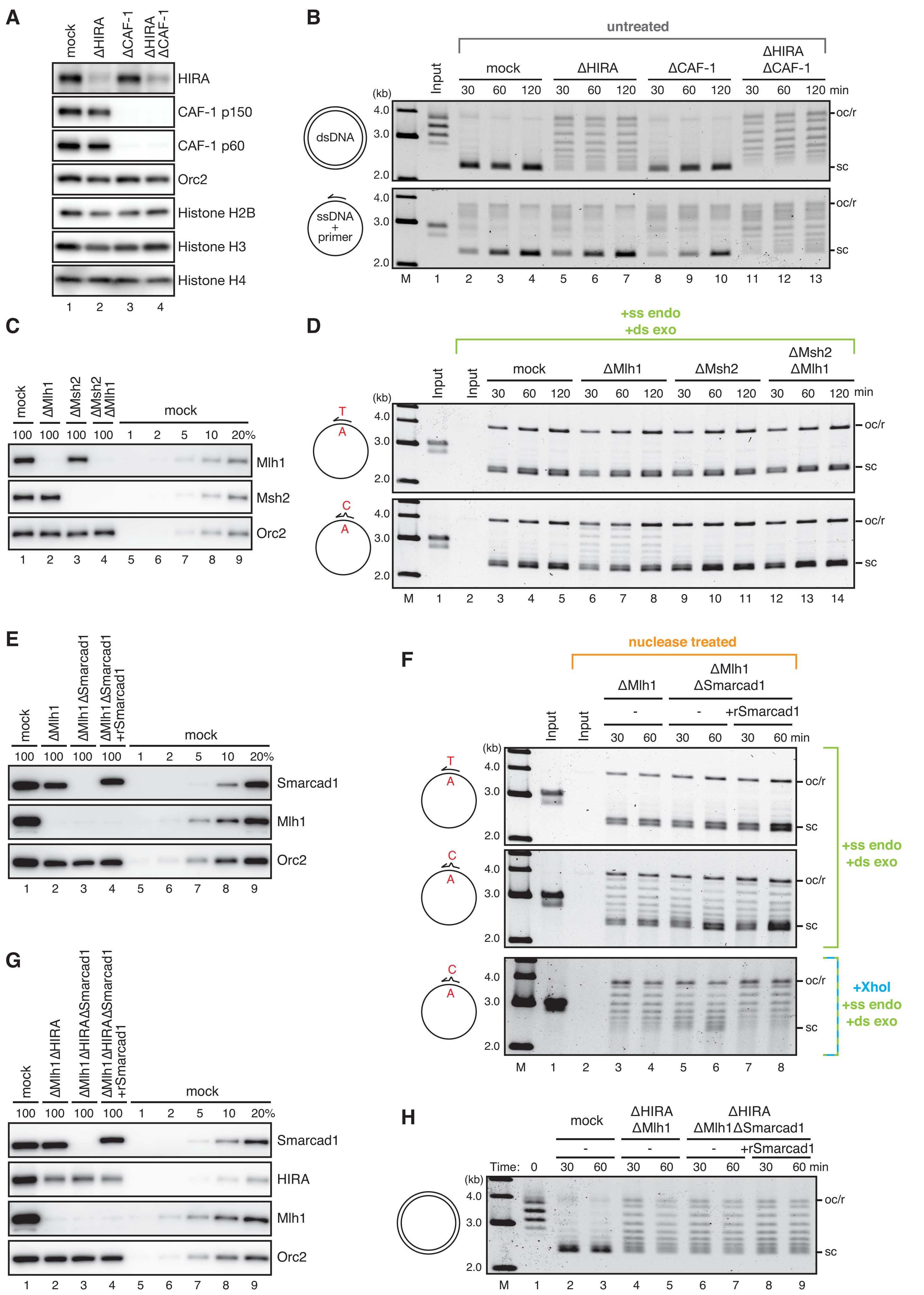

Terui_Supplemental Figure S6

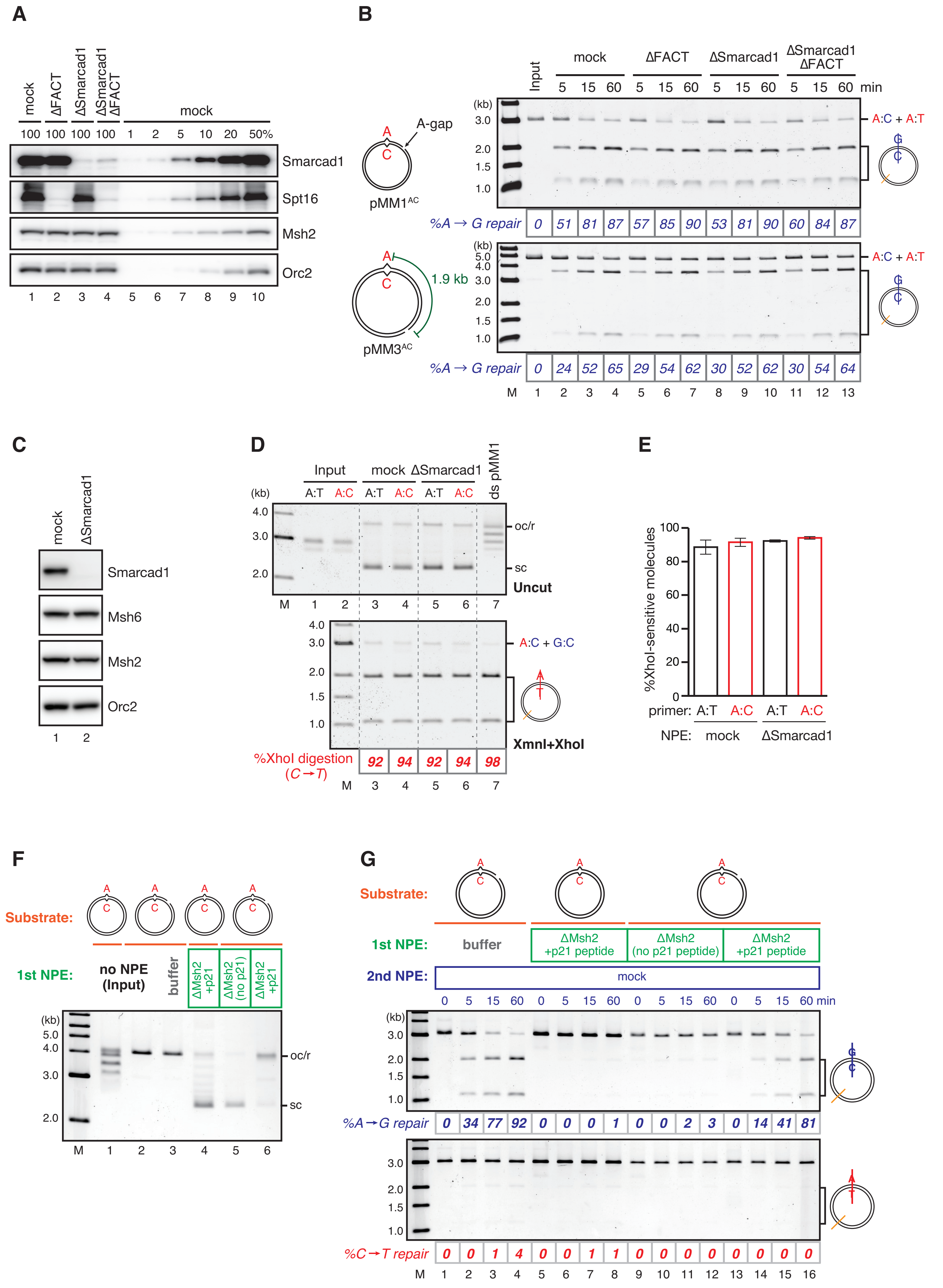

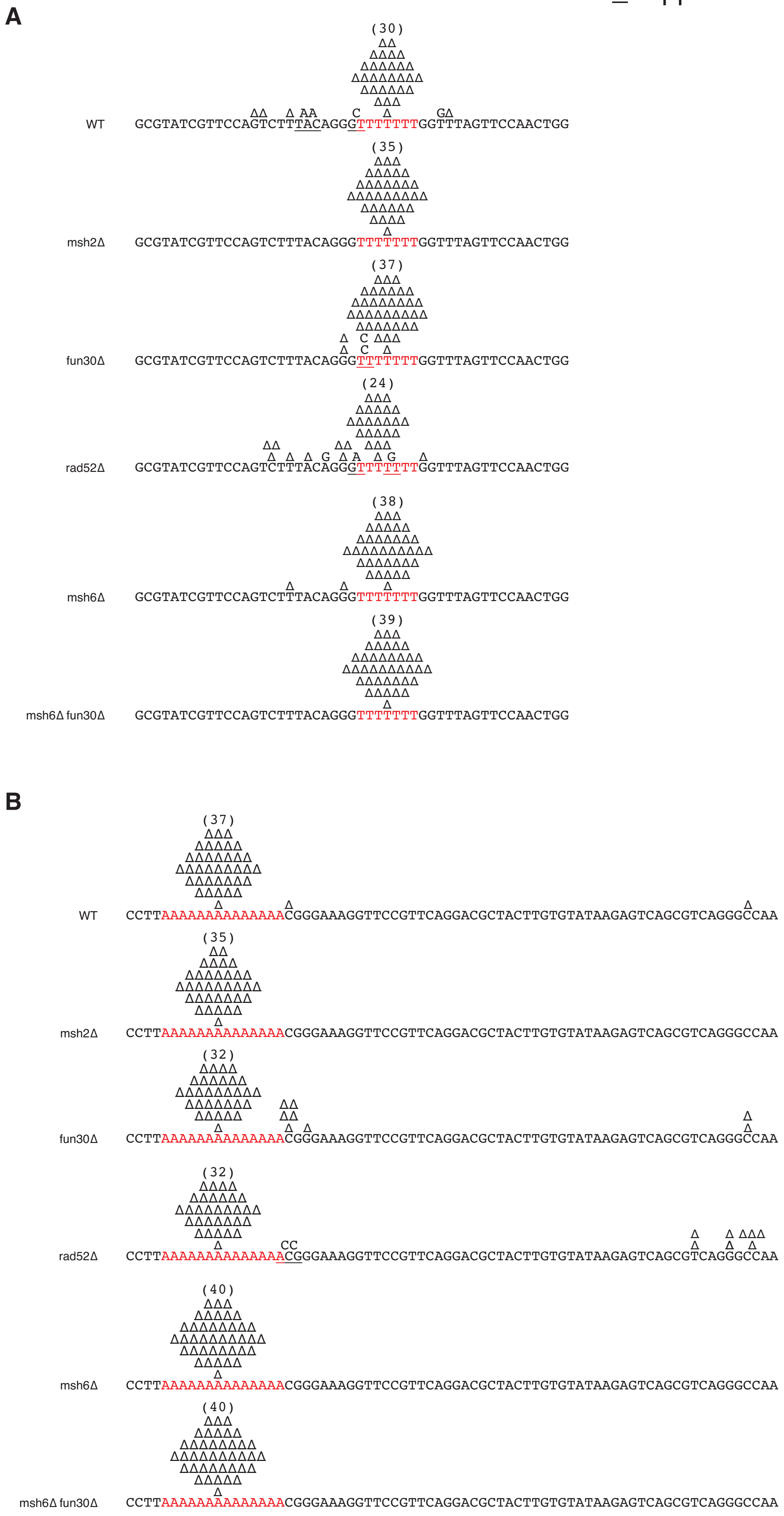
A
(30)
WT GCGTATCGTTCCAGTCTTTACAGGGTTTTTTTGGTTTAGTTCCAACTGG
(35)
msh2Δ GCGTATCGTTCCAGTCTTTACAGGGTTTTTTTGGTTTAGTTCCAACTGG
(37)
fun30Δ GCGTATCGTTCCAGTCTTTACAGGGTTTTTTTGGTTTAGTTCCAACTGG
(24)
rad52Δ GCGTATCGTTCCAGTCTTTACAGGGTTTTTTTGGTTTAGTTCCAACTGG
(38)
msh6Δ GCGTATCGTTCCAGTCTTTACAGGGTTTTTTTGGTTTAGTTCCAACTGG
(39)
msh6Δ fun30Δ GCGTATCGTTCCAGTCTTTACAGGGTTTTTTTGGTTTAGTTCCAACTGG
B
(37)
WT CCTTAAAAAAAAAAAAAACGGGAAAGGTTCCGTTCAGGACGCTACTTGTGTATAAGAGTCAGCGTCAGGGCCAA
(35)
msh2Δ CCTTAAAAAAAAAAAAAACGGGAAAGGTTCCGTTCAGGACGCTACTTGTGTATAAGAGTCAGCGTCAGGGCCAA
(32)
fun30Δ CCTTAAAAAAAAAAAAAACGGGAAAGGTTCCGTTCAGGACGCTACTTGTGTATAAGAGTCAGCGTCAGGGCCAA
(32)
rad52Δ CCTTAAAAAAAAAAAAAACGGGAAAGGTTCCGTTCAGGACGCTACTTGTGTATAAGAGTCAGCGTCAGGGCCAA
(40)
msh6Δ CCTTAAAAAAAAAAAAAACGGGAAAGGTTCCGTTCAGGACGCTACTTGTGTATAAGAGTCAGCGTCAGGGCCAA
(40)
msh6Δ fun30Δ CCTTAAAAAAAAAAAAAACGGGAAAGGTTCCGTTCAGGACGCTACTTGTGTATAAGAGTCAGCGTCAGGGCCAA

Supplemental Table S2. The effect of *rad52* deletion on reversion rates.

Genotype	*hom3-10*		*lys2-14A*	
	Reversion Rate ($\times 10^{-9}$)	(Fold increase)	Reversion Rate ($\times 10^{-7}$)	(Fold increase)
Wild-Type	1.6 [0.9 - 2.4]	(1.0)	0.37 [0.30 - 0.46]	(1.0)
fun30Δ	3.3 [2.2 - 4.7]	(2.1)	0.70 [0.58 - 0.82]	(1.9)
msh6Δ	10 [7.0 - 14]	(6.5)	69 [56 - 84]	(190)
msh6Δ fun30Δ	120 [100 - 150]	(79)	400 [350 - 450]	(1,100)
rad52Δ	15 [11 - 20]	(9.7)	1.3 [1.1 - 1.6]	(3.6)
rad52Δ fun30Δ	14 [10 - 18]	(8.8)	1.6 [1.3 - 1.8]	(4.2)
rad52Δ msh6Δ	44 [33 - 57]	(28)	120 [100 - 140]	(330)
rad52Δ msh6Δ fun30Δ	290 [250 - 340]	(190)	370 [320 - 430]	(1,000)

The *hom3-10* (Thr^+) and *lys2::insE-A14* (Lys^+) reversion rates are presented with 95% confidence intervals in square brackets. Fold increase of reversion rates over that of wild-type cells is shown in parentheses. The rates of *RAD52* cells were duplicated from Table 1.

Supplemental Table S3. The effect of *spt16-d922* mutation on reversion rates.

Genotype	*hom3-10*		*lys2-14A*	
	Reversion Rate ($\times10^{-9}$)	(Fold increase)	Reversion Rate ($\times10^{-7}$)	(Fold increase)
Wild-Type	1.6 [0.9 - 2.4]	(1.0)	0.37 [0.30 - 0.46]	(1.0)
fun30Δ	3.3 [2.2 - 4.7]	(2.1)	0.70 [0.58 - 0.82]	(1.9)
spt16-d922	1.9 [1.0 - 2.9]	(1.2)	0.15 [0.10 - 0.20]	(0.4)
spt16-d922 fun30Δ	2.8 [1.7 - 4.1]	(1.8)	0.54 [0.43 - 0.65]	(1.4)
msh6Δ	10 [7.0 - 14]	(6.5)	69 [56 - 84]	(190)
msh6Δ fun30Δ	120 [100 - 150]	(79)	400 [350 - 450]	(1,100)
msh6Δ spt16-d922	7.2 [5.0 – 9.6]	(4.5)	75 [63 - 87]	(200)
msh6Δ spt16-d922 fun30Δ	72 [56 - 90]	(46)	394 [344 - 448]	(1,100)

The *hom3-10* (Thr^+) and *lys2::insE-A14* (Lys^+) reversion rates are presented with 95% confidence intervals in square brackets. Fold increase of reversion rates over that of wild-type cells is shown in parentheses. The rates of *SPT16* cells were duplicated from Table 1.

Supplemental Table S4. ***S. cerevisiae*** **strains used in this study.**

Name	Relevant genotype	Parental strain	Reference
BY4741	*MATa his3Δ1 leu2Δ0 met15Δ0 ura3Δ0*	S288C	Brachmann et al., 1998
TTY15	BY4741 *hom3Δ::URA3*	BY4741	This study
TTY20	BY4741 *hom3-10*	TTY15	This study
TTY49	BY4741 *hom3-10 lys2::(YIplac211-URA3-lys2::insE-A14)*	TTY20	This study
TTY53	BY4741 *hom3-10 lys2::insE-A14*	TTY49	This study
TTY57	TTY53 *fun30Δ::kanMX*	TTY53	This study
TTY61	TTY53 *msh2Δ::kanMX*	TTY53	This study
TTY65	TTY53 *msh6Δ::kanMX*	TTY53	This study
TTY69	TTY53 *msh3Δ::kanMX*	TTY53	This study
TTY78	TTY53 *rad52Δ::hphMX6*	TTY53	This study
TTY82	TTY53 *fun30Δ::kanMX rad52Δ::hphMX6*	TTY57	This study
TTY86	TTY53 *fun30Δ::kanMX msh2Δ::hphMX6*	TTY57	This study
TTY88	TTY53 *fun30Δ::kanMX msh3Δ::hphMX6*	TTY57	This study
TTY90	TTY53 *fun30Δ::kanMX msh6Δ::hphMX6*	TTY57	This study
TTY96	TTY53 *exo1Δ::hphMX6*	TTY53	This study
TTY98	TTY53 *fun30Δ::kanMX exo1Δ::hphMX6*	TTY57	This study
TTY117	TTY53 *cac1Δ::natMX6*	TTY53	This study
TTY119	TTY53 *msh6Δ::kanMX cac1Δ::natMX6*	TTY65	This study
TTY121	TTY53 *fun30Δ::kanMX msh6Δ::hphMX6 cac1Δ::natMX6*	TTY90	This study
TTY136	TTY53 *msh2Δ::hphMX6 cac1Δ::natMX6*	TTY117	This study
TTY154	TTY53 *fun30-K603A*	TTY53	This study
TTY156	TTY53 *msh6Δ::kanMX fun30-K603A*	TTY65	This study
TTY164	TTY53 *spt16-d922*	TTY53	This study
TTY166	TTY53 *msh6Δ::kanMX spt16-d922*	TTY65	This study
TTY186	TTY53 *spt16-d922 fun30Δ::hphMX6*	TTY164	This study
TTY188	TTY53 *msh6Δ::kanMX spt16-d922 fun30Δ::hphMX6*	TTY166	This study
TTY198	TTY53 *fun30Δ::kanMX cac1Δ::natMX6*	TTY57	This study
TTY262	TTY53 *msh3Δ::kanMX cac1Δ::natMX6*	TTY69	This study
TTY264	TTY53 *fun30Δ::kanMX msh3Δ::hphMX6 cac1Δ::natMX6*	TTY88	This study

TTY296	TTY53 *msh6Δ::kanMX rad52Δ::natMX6*	TTY90	This study
TTY298	TTY53 *fun30Δ::kanMX msh6Δ::hphMX6 rad52Δ::natMX6*	TTY90	This study
TTY306	TTY53 *msh6Δ::kanMX exo1Δ::natMX6*	TTY65	This study
TTY308	TTY53 *fun30Δ::kanMX msh6Δ::hphMX6 exo1Δ::natMX6*	TTY90	This study

All strains were derived from S288C (BY4741).

Supplemental Table S5. Oligonucleotides used in this study.

No.	Sequence (5′ to 3′)
60	TATGGATTATAAAGATGATGACGATAAGGACTACAAGGATGACGACGATAAACTGGAAGTTCTGTTCCAGGGGCCCCT
61	TAAGGGGCCCCTGGAACAGAACTTCCAGTTTATCGTCGTCATCCTTGTAGTCCTTATCGTCATCATCTTTATAATCCA
81	GGAAAGCCATGGGCCACCACCACCATCACCATGATTATAAAGATGATGACGATAAGCTGG
344	GGGGACAAGTTTGTACAAAAAAGCAGGCTCCAC
345	GGGGACCACTTTGTACAAGAAAGCTGGGTC
362	CGCCTTGATCGT[Bio-dT]GGGAACCGGAGCTGAATGAAGC
406	GGGAACAAAAGCTGGGTACGAATTCCTGCAGCAGTTCGTGACTGTTAGCGACTGACGCAGTAACATGGATCTCGAGAT GCAGTACGGTCACC
407	GGGAACAAAAGCTGGGTACGAATTCCTGCAGCAGTTCGTGACTGTTAGCGACTGACGCAGTAACATGGATCCCGAGAT GCAGTACGGTCACC
411	CAGTAACATGGATCCGAGATGCAGTACGGTCACC
414	CAGTAACATGGATCTGGAGATGCAGTACGGTCACC
415	CAGTAACATGGATCTCGCGATGCAGTACGGTCACC
721	CAGTAACATGGATCTCGAGATGCAGTACGGTCACC
722	CAGTAACATGGATCCCGAGATGCAGTACGGTCACC
723	GGTCGGGCTGAACGGGGGGTTCGTGCACACAGCCC
724	GGTCGGGCTGAACGAGGGGTTCGTGCACACAGCCC
725	GGTGGCACTTTTCGGGGAAATGTGCGCGGAACCC
726	GGTGGCACTTTTCGTGGAAATGTGCGCGGAACCC
770	GGAACCATGGCGGATACGTTGGAG
771	GGAACCTGCAGGTTAATCTGAACCCGATTCAGCTGAGC
780	CTGCTGCCAGTTTACGGGATGATGACCG
784	CTTGTCTCCAGCTCAGTGCGGGATTTGG
798	AAAGCAGGCTCCACCATGGCGGTCACACTTGACAAAGAAGC
799	ACAAGAAAGCTGGGTCCTCGAGTCACTTCTTCCTCTTTTTAGAAGGATTTGG
827	GATGATGACGATAAGCTGGAAGTTCTGTTCCAGGGGCCCGGGATGGCGGTCACACTTGAC
887	ACAAGAAAGCTGGGTCGGATCCTTAAAGACCCAGGGATGTTTTC
900	AAAGCAGGCTCCACATATGTCAGCATTTAATCTGGAACGCTTCC
955	GCAACTGTACAAGCCATAGC
956	GCTATGGCTTGTACAGTTGCTCCCAGACCCATTTCATCTGC
957	AAAGCAGGCTCCACTCGAGGGATGCGGCGGCAGAAGCAGG
958	ACAAGAAAGCTGGGTGGATCCTCACTCCGTTATCCGCAACCATTC
1079	GGAACCTGCAGGAGCTTATACAGCTATCTTGACTCCCG
1158	GAGGAGCGGAAGAGCGGTGGCTTCTAGTAGCGATGTCGGGTAGA
1290	GTTCCGCCCGTTCTTCATACACC
1293	CTGCATCTGGAATGGGCAAAGCC
1294	CTGCGGCCGCACTCCAATTCGCCCTATAGTGAGTCG
1295	CCACCTGCAGGAGGTCCAGCTTTTGTTCCCTTTAGTGAGG
1296	TGGAGTGCGGCCGCAGTTGGCTAGCTATGCTATGCCTTCC
1297	GGACCTCCTGCAGGTGGCGGAATTTTCGATATCTGGAATCTTACC
1298	TTTTTTTTCCAGGATCTGACGTGGGCAAACACTTTGAAACTGTAG
1300	ACGGGAAAGGTTCCGTTCAGGACGCTACTTGTGTATAAGAGTCAG
1301	CTACTTGTGTATAAGAGTCAGCGTCAGGGCCAAGGATGAAGAAGC
1304	CCAGTTGGAACTAAACCAAAAAAACCCTGTAAAGACTGGAACG
1305	CGTTCCAGTCTTTACAGGGTTTTTTTGGTTTAGTTCCAACTGG
1311	AAAAATGCCGACAATGAGCC
1314	CGCCTCCAATGATTTCTTCC
1315	TTCGGTGGTACATCTGTCGG
1318	ACATGGTACCAGCAATGCCG
1347	TCGGTTCTTACTGCCAAGTG
1348	CGTCAGGTGGCACTTTTCGGCTGAGGATTTTGATGTTAGGTCAGC
1349	CGTCTCGCGCGTTTCGGTGATTCATAATCCCTAAAAGTGTTCACG
1350	GCAACGTTATCATGCTTTGAACG
1353	TGGAGTGCGGCCGCAGTGCGCTCATCGTTGGAATTC
1354	CGTCAGGTGGCACTTTTCGGACGTTCCTTACTGTAGATAGTCGGG
1355	CGTCTCGCGCGTTTCGGTGATCTTCTGCGTGAACGCACCATGTGG
1356	GGACCTCCTGCAGGTGGGTACATGTTAGGCCTGTCGG
1359	AACCTAAATGCGGGTTTTCG
1360	CGTCAGGTGGCACTTTTCGGTTTGGAGTTCAAATTGGCTTTGTC
1361	CGTCTCGCGCGTTTCGGTGATGGTCGTTGAAAAAGGATTCAGAAC
1362	GCGCATTCTTCTAAGTTCGG
1365	AGGTCTGTGCTTTTCTTCGG
1366	CGTCAGGTGGCACTTTTCGGTCTATTTGCACTGCTTTTGGCTCTC
1367	CGTCTCGCGCGTTTCGGTGACGGATGGTACGTATGTTCTAAATGC
1368	GGTTCCAAATCAGGAGCTGC
1426	CCTGAACGGAACCTTTCCCGTTTTTTTTTTTTTTTAAGGATCTGACGTGGGCAAACAC
1428	CCAAGCCAGCATCTGTATCACCAG
1429	CCACCAGTGAGCCCGCTTGCAG
1457	TGCCCATGCTATAGACTCCC

1458	CGTCAGGTGGCACTTTTCGGCAACAACACACCAAAGCCACC
1459	CGTCTCGCGCGTTTCGGTGAACGCTTCCTGGCCGAAAC
1460	GCATAGTTCAATTGCGTGACATC
1481	CATCACAGTTCATTGCTTCG
1482	CGTCAGGTGGCACTTTTCGGTCCTTTCTACGCCTTTCAG
1483	CGTCTCGCGCGTTTCGGTGATGCACGTTTCATATCGGAGG
1484	CAACGAGACGGTTCCGTTCC
1537	CCGGCGCCCAGTCAGCCTTTACG
1538	CGTCAGGTGGCACTTTTCGGCCATTTTCCCTTTTCCTTTCGATTCTC
1539	CGTCTCGCGCGTTTCGGTGACAACCCCGTCTTTGTAACTTGAACGG
1540	AGGCACAACGGGGGAGCTGGAG
1564	GGAAGAATTCGCACCAGCACCAGCACCAGTGG
1565	GACTTGACATGTAGCACCTAGACCCATGTCGTCTGCAAGG
1566	CATGGGTCTAGGTGCTACATGTCAAGTCATTTCATTTTTCGC
1567	GGAAGGATCCTTCCCAGATTGCATCCACTCGTC
1571	GGAAGGATCCCATTCCGAACCATGTGGTGCT
1572	GGCCATTTCTTCAGATGGATCGATATTCAGCTCTTCCATATTCACTTAAATACACG
1573	GTATTTAAGTGAATATGGAAGAGCTGAATATCGATCCATCTGAAGAAATGGCCAAC
1574	GGATTGACCTCGTCTTCCACCTCC
1840	GCTTAGTCTGTTCCATGTCATGC
1841	TGGGTACGAATTCCTGCAGC
1842	CGCAGAAGTGGTCCTGCAAC
1843	GACACCACGATGCCTGTAGC
1847	GTCGGGAAACCTGTCGTGCC
1848	CAGCGAGTCAGTGAGCGAGG
1862	TCATTGCGCTGCCATTCTCC
1864	GAAACTTCGGCTTCCCCTGG
1876	CATGTGAGCAAAAGGCCAGC
1877	GTTTCGCCACCTCTGACTTG
1878	CTCATAGCTCACGCTGTAGG
1879	ACCGGGTTGGACTCAAGACG
1916	TTTCCCCGAAAAGTGCCACC
1917	GGAAGGGAAGAAAGCGAAAGG
1918	GCCGGCTTTCCCCGTCAAGC
1919	CAGGGCGATGGCCCACTACG
1920	GCTGTATAAGCTCCTGCAGC
1921	CACTTATCTTCCCCGAAGCC
1922	AGATTTGTTTGGAGAGTGCAGG
1923	ATAGCAATCCCACTTGTGTCAAG
1924	ACTTTGCGCGTTAGGATGTAGC
1925	AACAAGAGCGCTTGGTTGTC
1926	CTTCATCCTATTCCGTTACGCG
